# Efficient and secure three-party mutual authentication key agreement protocol for WSNs in IoT environments

**DOI:** 10.1371/journal.pone.0232277

**Published:** 2020-04-30

**Authors:** Chi-Tung Chen, Cheng-Chi Lee, Iuon-Chang Lin

**Affiliations:** 1 Department of Distribution Management (Information Management), National Chin-Yi University of Technology, Taichung, Taiwan, China; 2 Department of Library and Information Science, Research and Development Center for Physical Education, Health, and Information Technology, Fu Jen Catholic University, New Taipei City, Taiwan, China; 3 Department of Photonics and Communication Engineering, Asia University, Taichung, Taiwan, China; 4 Department of Management Information Systems, National Chung Hsing University, Taichung, Taiwan, China; Wuhan University, CHINA

## Abstract

In the Internet of Things (IoT), numerous devices can interact with each other over the Internet. A wide range of IoT applications have already been deployed, such as transportation systems, healthcare systems, smart buildings, smart factories, and smart cities. Wireless sensor networks (WSNs) play crucial roles in these IoT applications. Researchers have published effective (but not entirely secure) approaches for merging WSNs into IoT environments. In IoT environments, the security effectiveness of remote user authentication is crucial for information transmission. Computational efficiency and energy consumption are crucial because the energy available to any WSN is limited. This paper proposes a notably efficient and secure authentication scheme based on temporal credential and dynamic ID for WSNs in IoT environments. The Burrows–Abadi–Needham (BAN) logic method was used to validate our scheme. Cryptanalysis revealed that our scheme can overcome the security weaknesses of previously published schemes. The security functionalities and performance efficiency of our scheme are compared with those of previous related schemes. The result demonstrates that our scheme’s security functionalities are quantitatively and qualitatively superior to those of comparable schemes. Our scheme can improve the effectiveness of authentication in IoT environments. Notably, our scheme has superior performance efficiency, low computational cost, frugal energy consumption, and low communication cost.

## 1. Introduction

**Fig 1:**
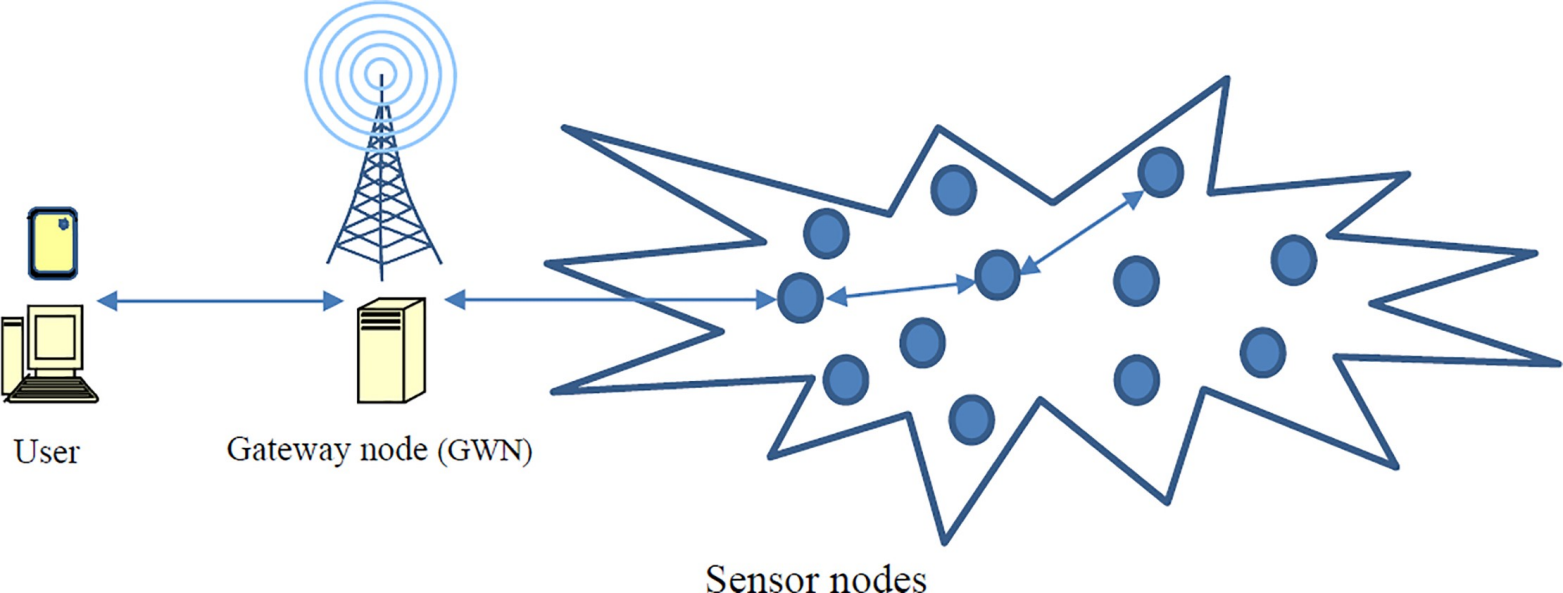
Wireless sensor networks.

Internet of Things (IoT) is an emerging technology, which is the extension of Internet connectivity into various devices such as sensors, vehicles, and mobile phones. These devices can interact with each other over the Internet [1]. A wide range of applications connecting objects that can communicate with each other have been deployed; applications include transportation systems, healthcare systems, smart buildings, smart factories, and smart cities [1, 2]. Wireless sensor networks (WSNs) are crucial in these IoT applications [2, 3]. WSNs have become increasingly used in providing services for monitoring environments and activities because of their low cost, flexibility, ease of deployment, and wide range of applications (Fig 1) [4]. As illustrated in Fig 1, WSNs comprise numerous sensor nodes scattered arbitrarily over a certain region. Sensor nodes can sense, process, and transmit information (e.g., temperature and traffic information). Remote users are required to reach a specific sensor node via the gateway node (GWN) [5, 6]. Each scattered sensor node can collect data and route data back to the GWN. Remote users may communicate with a GWN through the Internet. When data from WSNs are made available to users, the legitimacy of each user must be verified before the system can grant access to the data, and the sensor nodes reserved for access must be confirmed to be legitimate. Hence, remote user authentication is necessary and critical for secure information transmission in WSNs [2, 5, 7, 8]. The following basic design criteria must be considered when designing a remote user authentication scheme for WSNs [2, 5, 8]:

1. *Mutual authentication*. Users and sensor nodes must mutually authenticate each other. After they have authenticated each other, they must arrange a session key for information transmission.
2. *Masquerade attack resistance*. An adversary cannot impersonate a legal user to log in to WSNs. In addition, the adversary cannot masquerade as a sensor node to spoof the user.
3. *Replay attack resistance*. The adversary cannot attempt to replay previously intercepted messages to spoof the GWN.
4. *Guessing attack resistance*. The adversary cannot obtain useful information to devise an offline check of the correctness of guessed passwords.

### 1.1. Preliminaries and technical background

In this subsection, we introduce some preliminaries and the principal technologies that our scheme is based on, such as temporal credential [7] and dynamic ID [9, 10].

A temporal credential is an impermanent attestation of authority issued by a third party. The GWN can issue a temporal credential to each user and sensor node [7]. The expiration time of a user’s temporal credential is regulated by the GWN. A user’s temporal credential is related to the identity of user and can be securely stored in a smart card. The temporal credential of a sensor node is also related to its identity and confidentially written in its storage. Based on the issuing and signing of temporal credential, the mutual authentication between the user and the GWN is achieved through the verification of temporal credential for the user. The mutual authentication between the sensor node and the GWN is achieved by the verification of the temporal credential for the sensor node.

Each dynamic ID is temporarily assigned by the system and mapped to a specific user [9]. A dynamic ID is a combination of its user’s information and a random nonce. The random nonce is an arbitrary number; it is used only once during the communication. In the authentication process, the login message of the user *i* contains a dynamic ID, called *DID*_*i*_. The login message is dynamic for each login. For all *i*, the parameter *DID*_*i*_ is associated with nonce *N*_*i*_ and changed dynamically for each login. The use of a dynamic ID in each login message can avoid the risk of ID-theft [10]. Our scheme introduces dynamic ID to anonymize users.

### 1.2. Motivation and contribution

Typical IoT installations allow remote users to access data from sensor nodes in WSNs through the Internet. Researchers have been developing effective approaches for merging WSNs into IoT environments [2, 11–16]. Because of the resource constraints of sensor nodes, to design an efficient and secure authentication scheme for WSNs in IoT environments constitutes a nontrivial challenge. In IoT environments, the security effectiveness of remote user authentication is crucial for trustworthy information transmission [2, 3]. Computational efficiency and energy consumption are crucial because of the limited energy resources of WSNs [2, 3]. Moreover, time synchronization is a critical and challenging problem for WSNs; the system must provide a synchronized logical time clock for all devices and objects in IoT environments [3, 17–19]. Any adversary and any malicious node in IoT environments can attack clock synchronization [3, 17]. The communication errors, frequent topological changes, low-cost clocks, and limited energy levels of IoT nodes are other factors that can affect time synchronization [18, 19]. A timestamp-based authentication scheme requires trustworthy timestamps and synchronized time clocks to verify any device’s legitimacy. When a system has a serious time synchronization problem, no device can be synchronized with any another device, and thus the system cannot verify any device’s legitimacy. Therefore, any serious time synchronization failure causes mutual authentication failure. The time synchronization problem should be contemplated as designing a remote user authentication scheme for WSNs in IoT environments [3, 17–19]. Moreover, when a given user’s ID is revealed, an adversary can determine any information concerning the user's identity and monitor the user’s activities. An exposed user ID is also useful to the adversary because it provides login information [10]. Therefore, anonymous access for each login should be required. Although several previously published studies have proposed diverse remote user authentication schemes, they have been neither highly secure nor efficient sufficiently to satisfy the requirements of WSNs in IoT environments (Related work in Section 2). This paper proposes a more efficient and secure authentication scheme for WSNs in IoT environments to ameliorate these security weaknesses.

The major contributions of our work are as follows:

1. We propose a new three-party scheme on the basis of temporal credential [7] and dynamic ID [9, 10] for WSNs in IoT environments to achieve security, mutual authentication, and session key agreement. Cryptanalysis revealed that the security functionalities of the proposed scheme qualitatively and quantitatively superior to those of previous schemes; the proposed scheme can advance the field of authentication schemes. The Burrows–Abadi–Needham (BAN) logic method [3, 20–24] was used to validate our scheme.
2. The proposed scheme performs efficiently in IoT environments, with low computational cost, frugal energy consumption, and little communication cost.
3. Our scheme uses temporal credentials and random nonce instead of the timestamps to verify mutual authentication among *U*_*i*_, the *GWN*, and *S*_*j*_. Therefore, our scheme can avoid the time synchronization problem for WSNs in IoT environments [3, 9, 17, 25]. Moreover, dynamic ID technology [9, 10] is applied in our scheme. User identities are consequently anonymous and can be confirmed only by the service provider.

### 1.3. Organization of the paper

The remainder of this paper is organized as follows: Section 2 introduces a brief review of the related work in WSNs and explains the security weaknesses of the Ostad-Sharif et al. scheme [2] for WSNs in IoT environments; Section 3 details the proposed efficient secure authentication scheme for WSNs in IoT environments; Section 4 presents the security analysis of the proposed scheme; Section 5 discusses the effectiveness and efficiency of the proposed scheme; and finally, Section 6 presents the study’s conclusion.

## 2. Related work in WSNs

To satisfy the security requirements of WSNs, many remote user authentication schemes have been proposed. In 2004, Benenson et al. [26] described the security issues of user authentication in WSNs and proposed a protocol for them, in which the user can achieve successful authentication with any subset of sensors from a set of *n* sensors (*n* being the average number of sensors within a broadcast distance of the user). Watro et al. [27] proposed a TinyPK authentication protocol with the Rivest-Shamir-Adleman (RSA) public key cryptosystem [28] and Diffie-Hellman key agreement algorithm [29]. However, this authentication protocol has the disadvantage of the masquerade attack, in which an adversary can masquerade as a sensor node to spoof the user [5]. Wong et al. [30] proposed a less complex lightweight user authentication protocol for WSNs by using hash function operations. However, the scheme cannot protect against stolen-verifier, replay, and forgery attacks [5, 31]. Moreover, the passwords in the scheme can be revealed easily by any of the sensor nodes, and users cannot change their passwords freely. In 2009, to eliminate the weaknesses of the Wong et al. scheme, Das [5] proposed a two-factor user authentication scheme for WSNs. The scheme implements password-based authentication with the assistance of a GWN to access resource-constrained sensor nodes. However, this scheme is vulnerable to insider, masquerade, offline password-guessing, stolen smart card, and GWN bypassing attacks [7, 8, 32]. The scheme does not provide mutual authentication, a key agreement, and a password change phase for users to change or update their password [7, 8, 32]. Khan et al. [32], Chen et al. [33], and Yeh et al. [8] have subsequently proposed new schemes for improving the inherent security weaknesses of the Das scheme. Khan et al. [32] proposed a user authentication scheme for rectifying the susceptibilities of the Das scheme and achieving a more secure user authentication in WSNs. Afterward, Chen et al. [33] provided a secrecy-improved mutual user authentication scheme for WSNs by applying hash functions. Yeh et al. [8] proposed a new mutual user authentication protocol by using elliptic curves cryptography (ECC) and smart cards for WSNs. Xue et al. [7] showed that the Khan et al. scheme is vulnerable to stolen smart card and GWN bypassing attacks. In addition, the Chen et al. scheme is vulnerable to insider, masquerade, stolen smart card, and GWN bypassing attacks [7]. By contrast, the Yeh et al. scheme is vulnerable to stolen smart card and replay attacks [7]. Xue et al. [7] proposed a temporal-credential-based mutual authentication scheme for users, GWNs, and sensor nodes. With the assistance of password-based authentication, the GWN in the Xue et al. scheme can issue a temporal credential to each user and sensor node. However, the Xue et al. scheme is vulnerable to insider attacks and stolen smart card attacks [34]; the scheme does not offer password protection [34]. In 2016, Chang et al. [35] proposed a flexible authentication scheme for WSNs which operates in two modes. The first mode provides a lightweight authentication scheme, and the second mode is an advanced protocol based on ECC. In 2018, Amin et al. [34] demonstrated that the Chang et al. scheme is insecure against stolen smart card attack and cannot provide password protection. Amin et al. [34] then proposed a robust authentication scheme using smartcards for WSNs. However, the Amin et al. scheme has higher energy consumption, computational costs, and communication costs than those published previously (Section 5) [34]. In healthcare applications, Challa et al. [36] proposed a secure user authentication scheme for wireless healthcare sensor networks. The three factor authentication scheme is designed with ECC. The proposed scheme has several functionality features including dynamic sensor node addition, password updates, biometrics updates, and smart card revocation for WSNs. On the basis of ECC, Li et al. [3] also proposed an anonymous authentication scheme for WSNs in IoT environments. In the scheme, they used fuzzy commitment scheme [3] to handle user biometric information. In 2019, Harbi et al. [37] proposed an ECC-based mutual authentication scheme to secure communication in IoT-enabled WSNs. The sensor network in the system is arranged into clusters to diminish the energy consumption of sensors. Each cluster has a cluster head, which is a leader sensor node. However, Challa et al. scheme, Li et al. scheme, and Harbi et al. scheme are all based on an ECC for WSNs. The ECC approach is a public key cryptography approach based on elliptic curves. According to a related study, the time cost of an ECC point multiplication is much larger than that of hash function operations [2, 3, 7, 34, 35], and the energy consumption for executing an asymmetric ECC cryptosystem is much higher than that for executing a hash function [38, 39].

Currently, researchers are designing effective remote user authentication schemes for WSNs in IoT environments. In 2019, Ostad-Sharif et al. [2] proposed an efficient user authentication scheme and claimed that their scheme is appropriate for WSNs in IoT environments. However, in this section, we argue that the login and authentication phase of the Ostad-Sharif et al. scheme has design faults. Moreover, their scheme cannot provide password change and update a password in its password change phase. Their scheme also has the time synchronization problem [3, 17–19]. The details are presented as follows.

### 2.1. Authentication design faults of the Ostad-Sharif et al. scheme in IoT environments

Design faults exist in the login and authentication phase of the Ostad-Sharif et al. scheme [2]. We illustrate this security weakness in the subsequent passages. When a registered user *U*_*i*_ wants to access the information of sensor node *S*_*j*_, the login and authentication phase of the Ostad-Sharif et al. scheme must be executed in advance. At first, a registered user *U*_*i*_ inserts a smart card into the smart card reader and imprints his/her fingerprint *B*_*i*_ on the sensor device. The smart card contains the secret parameters {*D*_*i*_, *C*_*i*_, *E*_*i*_, *SCN*_*i*_, *BK*()}, in which *SCN*_*i*_ denotes unique smart card number and *BK*() denotes biometric key generation/extraction function. The smart card reader first extracts masked biometric *C*_*i*_ from the smart card and computes *RN′*_*i*_ = *BK*(h(*B*_*i*_))⊕*C′*_*i*_. After finding *C′*_*i*_, the smart card reader must validate whether *C′*_*i*_ and *C*_*i*_ are equal. If *C′*_*i*_ ≠ *C*_*i*_, then the smart card reader terminates the request. However, in the equation above, the smart card reader does not know random number *RN′*_*i*_ and masked biometric *C′*_*i*_. Therefore, it cannot obtain *RN′*_*i*_ and *C′*_*i*_ from the equation. Finally, a legitimately registered user *U*_*i*_ cannot pass the verification to access the system. This problem will happen to all legitimately registered users. The Ostad-Sharif et al. scheme has design faults in the login and authentication phase.

### 2.2. Failure to provide password change capability in the Ostad-Sharif et al. scheme

The Ostad-Sharif et al. scheme [2] cannot provide password change capability. We demonstrate this weakness in the following passages. When a registered user *U*_*i*_ wants to update the password *PW*_*i*_, the password change phase in the scheme must be executed. *U*_*i*_ first inserts a smart card into the smart card reader. He or she then inputs identity *ID*_*i*_ and password *PW*_*i*_. The smart card contains the secret parameters {*D*_*i*_, *C*_*i*_, *E*_*i*_, *SCN*_*i*_, *BK*()}. After the legitimacy of *U*_*i*_ is verified, *U*_*i*_ enters a new password $PW_{i}^{new}$. The smart card computes the following equations:

1. $RPW_{i}^{new}$ = h(ID_i_∥ $PW_{i}^{new}$∥RN_i_), in which RN_i_ denotes random number.
2. *A′_i_* = *D_i_*⊕*RPW*_*i*_
3. $D_{i}^{new}$ = $A_{i}^{new}$ ⊕ *RPW*_*i*_
4. *L′_i_* = *E_i_* ⊕ *RPW*_*i*_
5. $E_{i}^{new}$ = L′_i_ ⊕ *RPW*_*i*_

After $D_{i}^{new}$ and $E_{i}^{new}$ have been found, the smart card replaces the secret parameters {*D*_*i*_, *E*_*i*_} in the smart card with the new parameters {$D_{i}^{new}$, $E_{i}^{new}$}. The smart card finally contains the parameters {$D_{i}^{new}$, *C*_*i*_*, $E_{i}^{new}$, SCN*_*i*_, *BK*()}. However, in (3), the smart card does not know $A_{i}^{new}$; hence, it cannot obtain the new parameter $D_{i}^{new}$ from (3). Moreover, from (4) and (5), we obtain the following results:

$$E_{i}^{new}=L_{i}^{\prime }⊕RPW_{i}=E_{i}⊕RPW_{i}⊕RPW_{i}=E_{i}$$

Finally, the value of the new parameter $E_{i}^{new}$ is the same as the value of the parameter *E*_*i*_, and the new parameter $D_{i}^{new}$ cannot be acquired from the equations. Therefore, a registered user *U*_*i*_ cannot update his/her password. The Ostad-Sharif et al. scheme fails to provide password change capability.

### 2.3. Time synchronization and authentication problem of the Ostad-Sharif et al. scheme in IoT environments

The Ostad-Sharif et al. scheme uses a timestamp *T*_*i*_ to verify mutual authentication among *U*_*i*_, the *GWN*, and *S*_*j*_ for WSNs in IoT environments. Therefore, the Ostad-Sharif et al. scheme must provide synchronized time clocks to all devices in IoT environments for timestamp comparison [3, 17, 18]. However, as mentioned, both adversaries and malicious nodes can attack time synchronization [17]. Frequent topological changes, low-cost clocks, and limited energy of the sensor nodes in IoT environments can also affect time synchronization [18, 19]. The time synchronization of all WSN devices in IoT environments is a nontrivial challenge in itself [3, 17–19]. When a serious time synchronization problem arises in Ostad-Sharif et al. scheme, the *GWN*, *U*_*i*_, and *S*_*j*_ cannot be synchronized with each other and then the legitimacy values of the *GWN*, *U*_*i*_, and *S*_*j*_ cannot be verified. Hence, the Ostad-Sharif et al. scheme may enter a state such that mutual authentication among the *GWN*, *U*_*i*_, and *S*_*j*_ cannot be achieved [3, 17, 18].

## 3. Proposed scheme

In this section, we propose an efficient and secure authentication scheme for WSNs in IoT environments. The WSN environment contains three participants: the user (*U*_*i*_), sensor node (*S*_*j*_), and gateway node (*GWN*). The scheme applies dynamic ID to achieve security and user anonymity (identity protection) [9, 10]. The scheme applies temporal credential to achieve mutual authentication and session key agreement [7]. Temporal credentials are securely protected and stored in smart cards. The scheme can withstand stolen smart card attacks (Section 4.2). The system protects passwords against off-line password guessing attacks (Section 4.2). The system need not maintain any password or verification table; therefore it can resist the stolen verifier attacks and insider attacks [9, 40, 41]. The scheme can withstand masquerade attacks, replay attacks, GWN bypassing attacks, and GWN spoofing attacks (Section 4.4 and 4.8). Before the registration, users are not obliged to share their IDs and passwords with the GWN; hence, the scheme provides a convenient functionality of adding new users (Section 4.6). To solve the password-changing problem in previous schemes, we also introduce a new password change phase to update the password. In the new password change phase, *U*_*i*_ can freely select and update the password without requiring the communication with any other participants (the *GWN* and *S*_*j*_), such that it can avoid additional communication message overhead (Fig 5) [42]. Hash function is operated in our scheme for providing security and computational efficiency. Table 1 lists the definition of the notations in our scheme. The *GWN* chooses the private keys *K*_*GWN-U*_ and *K*_*GWN-S*_, and only the *GWN* knows them. The proposed scheme consists of four phases: (1) registration phase, (2) login phase, (3) authentication and key agreement phase, and (4) password change phase. They are described as follows:

**Table 1:** Notation definitions.

Notation	Definition
*U*_*i*_	The *i*th user
*S*_*j*_	The *j*th sensor node
*GWN*	The gateway node
*ID*_*i*_	The identification of *U*_*i*_
*ID*_*GWN*_	The identification of the *GWN*
*SID*_*j*_	The identification of *S*_*j*_
*DID*_*i*_	The dynamic ID of *U*_*i*_
*DID*_*GWN*_	The dynamic ID of the *GWN*
*PW*_*i*_	The password of *U*_*i*_
*PW*_*j*_	The password of *S*_*j*_
*BK*	Biometric key generation/extraction function
*B*_*i*_	Biometric of *U*_*i*_
*SCN*_*i*_	Unique smart card number
*K*_*GWN-U*_	Private key only known to the GWN
*K*_*GWN-S*_	Private key only known to the GWN
*KEY*_*ij*_	Shard session key between *U*_*i*_ and *S*_*j*_
*TC*_*i*_	Temporal credential issued by the GWN to *U*_*i*_
*TC*_*j*_	Temporal credential issued by the GWN to *S*_*j*_
*TE*_*i*_	Expiration time of a user’s temporal credential
*TS*	Timestamp value
*||*	String concatenation manipulation
→	Common channel^a^
⊕	Exclusive-or manipulation
⇒	Secure channel^b^
h(•)	One-way hash function^c^

^a^ A common channel is a channel allocated in common to participants.

^b^ A secure channel is a channel of delivering messages that can withstand tampering and overhearing.

^c^ A hash function has a one-way property that it is computationally infeasible to find a data object to map to a hash result [43].

### 3.1. Registration phase

**Fig 2:**
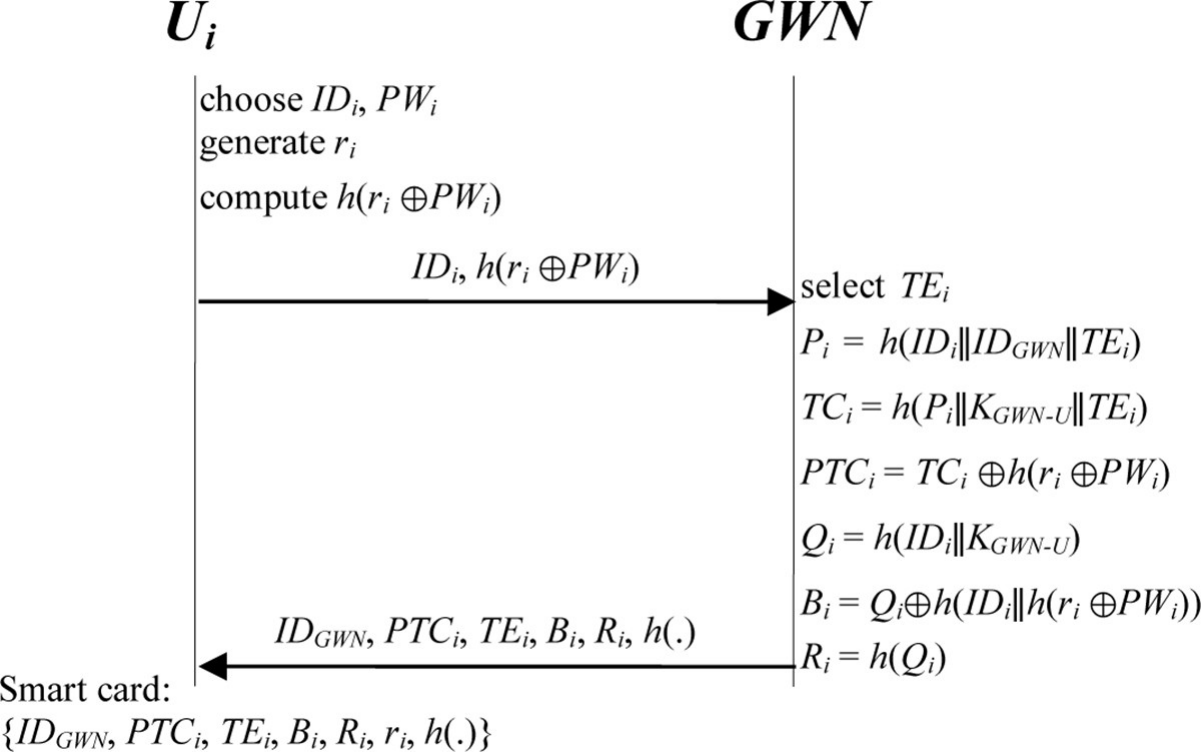
Registration phase for users in the proposed scheme.

The registration phase comprises two parts, one for users and the other for sensor nodes. We first describe the registration phase for users. In this phase, when a new user *U*_*i*_ undertakes to register, he or she selects the identification *ID*_*i*_ and password *PW*_*i*_. Subsequently, *U*_*i*_ generates a random number *r*_*i*_ and sends *ID*_*i*_ and *h*(*r*_*i*_⊕*PW*_*i*_) to the *GWN* for registration through a secure channel. After receiving the messages from *U*_*i*_, the *GWN* selects the expiration time *TE*_*i*_ of the temporal credential of *U*_*i*_. The *GWN* computes the temporal credential *TC*_*i*_ and verification information *R*_*i*_ for *U*_*i*_. The *GWN* then issues a smart card with the temporal credential *TC*_*i*_, expiration time *TE*_*i*_, and verification information *R*_*i*_ to *U*_*i*_ through a secure channel. The steps are detailed as follows (Fig 2):

**Step U1.** *U*_*i*_ freely chooses identification *ID*_*i*_ and password *PW*_*i*_.
**Step U2.** *U*_*i*_ generates a random number *r*_*i*_ and calculates *h*(*r*_*i*_⊕*PW*_*i*_).
**Step U3.** *U*_*i*_ ⇒ *GWN*: {*h*(*r*_*i*_⊕*PW*_*i*_), *ID*_*i*_}.

*U*_*i*_ transmits *h*(*r*_*i*_⊕*PW*_*i*_) and *ID*_*i*_ to the *GWN* through a secure channel.

**Step U4.** *GWN* ⇒ *U*_*i*_: {*ID*_*GWN*_, *PTC*_*i*_, *TE*_*i*_, *B*_*i*_, *R*_*i*_, *h*(.)}. After receiving the message from *U*_*i*_, the *GWN* selects the expiration time *TE*_*i*_ of the temporal credential of *U*_*i*_ and computes the following equations to issue the temporal credential *TC*_*i*_ for *U*_*i*_.

*P*_*i*_ *= h(ID*_*i*_*∥ID*_*GWN*_*∥TE*_*i*_*)*, *TC*_*i*_ *= h(P*_*i*_*∥K*_*GWN-U*_*∥TE*_*i*_*)*, *PTC*_*i*_ *= TC*_*i*_*⊕h(r*_*i*_*⊕PW*_*i*_*)*,

*Q*_*i*_ *= h(ID*_*i*_*∥K*_*GWN-U*_*)*, *B*_*i*_ *= Q*_*i*_*⊕h(ID*_*i*_*∥h(r*_*i*_*⊕PW*_*i*_*))*, *and R*_*i*_ *= h(Q*_*i*_*)*.

The *GWN* then issues a smart card with the secret parameters {*ID*_*GWN*_, *PTC*_*i*_, *TE*_*i*_, *B*_*i*_, *R*_*i*_, *h*(.)} to *U*_*i*_ through a secure channel.

**Step U5.** *U*_*i*_ stores *r*_*i*_ in the smart card, after which the smart card holds the parameters {*ID*_*GWN*_, *PTC*_*i*_, *TE*_*i*_, *B*_*i*_, *R*_*i*_, *r*_*i*_, *h*(.)}.

**Fig 3:**
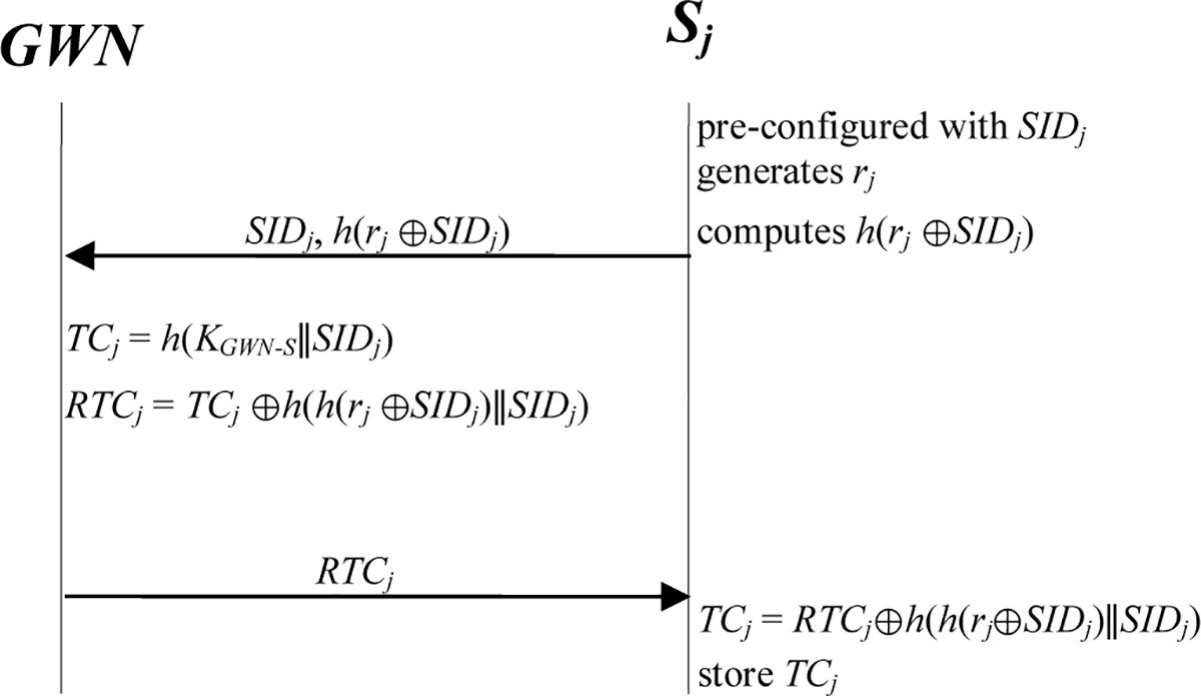
Registration phase for sensor nodes in the proposed scheme.

We now describe the registration phase for sensor nodes. In this phase, each sensor node *S*_*j*_ is pre-configured with *SID*_*j*_. After deployment, the sensor node *S*_*j*_ generates a random number *r*_*j*_ and then sends *SID*_*j*_ and *h*(*r*_*j*_⊕*SID*_*j*_) to the *GWN* for registration through a secure channel. After receiving the messages from *S*_*j*_, the *GWN* issues a temporal credential *TC*_*j*_ to *S*_*j*_ through a secure channel. The steps are detailed as follows (Fig 3):

**Step S1.** *S*_*j*_ is pre-configured with *SID*_*j*_.
**Step S2.** *S*_*j*_ generates a random number *r*_*j*_ and computes *h*(*r*_*j*_⊕*SID*_*j*_).
**Step S3.** *S*_*j*_ ⇒ *GWN*: {*SID*_*j*_, *h*(*r*_*j*_⊕*SID*_*j*_)}.

*S*_*j*_ sends *SID*_*j*_ and *h*(*r*_*j*_⊕*SID*_*j*_) to the *GWN* through a secure channel.

**Step S4.** *GWN* ⇒ *S*_*j*_: {*RTC*_*j*_}. After receiving the message from *S*_*j*_, the *GWN* computes *TC*_*j*_ = *h*(*K*_*GWN-S*_∥*SID*_*j*_) to issue the temporal credential *TC*_*j*_ for *S*_*j*_ and then calculates *RTC*_*j*_ = *TC*_*j*_⊕*h*(*h*(*r*_*j*_⊕*SID*_*j*_)∥*SID*_*j*_). The *GWN* sends *RTC*_*j*_ to *S*_*j*_ through a secure channel.
**Step S5.** After receiving the message from the *GWN*, *S*_*j*_ computes *TC*_*j*_ = *RTC*_*j*_⊕*h*(*h*(*r*_*j*_⊕*SID*_*j*_)∥*SID*_*j*_) to find its temporal credential *TC*_*j*_ and then stores it.

### 3.2. Login phase

**Fig 4:**
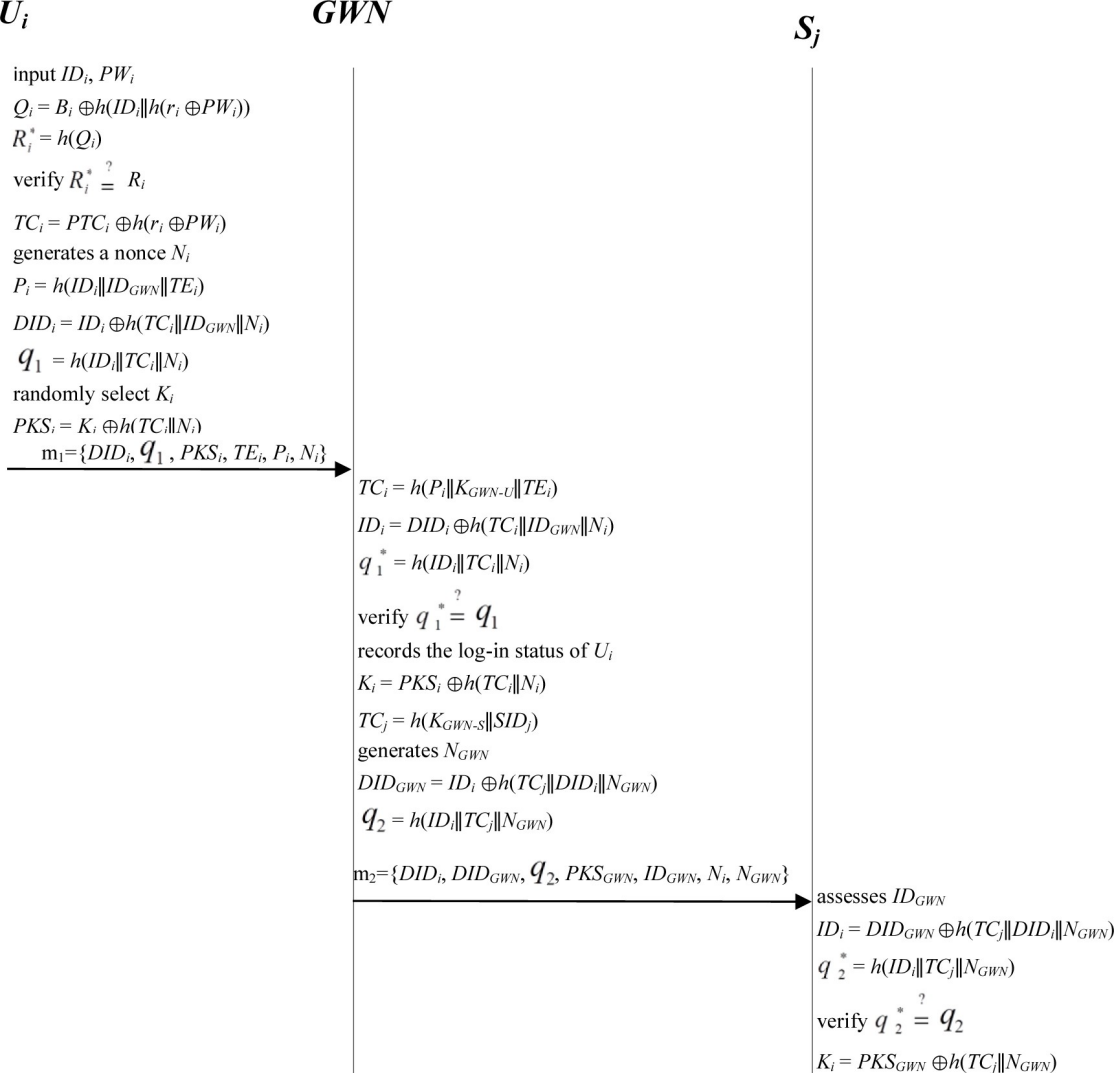
Login phase; authentication and key agreement phase.

*U*_*i*_ first inserts a smart card into the smart card reader to log in to the system. *U*_*i*_ then gives (*ID*_*i*_, *PW*_*i*_) that correspond to the smart card. The smart card of *U*_*i*_ computes verification information $R_{i}^{*}$ and then verifies it with the stored *R*_*i*_ in the smart card. After passing verification, the legitimacy of *U*_*i*_ is ensured. Afterward, *U*_*i*_ can read the information stored in the smart card and find its temporal credential *TC*_*i*_. The steps are detailed as follows (Fig 4):

**Step L1.** User *U*_*i*_ inserts a smart card into the smart card reader and provides keys (*ID*_*i*_, *PW*_*i*_). The smart card of user *U*_*i*_ then computes *Q*_*i*_ = *B*_*i*_⊕*h*(*ID*_*i*_∥*h*(*r*_*i*_⊕*PW*_*i*_)) and $R_{i}^{*}$ = *h*(*Q*_*i*_). The smart card validates whether $R_{i}^{*}$ and the stored *R*_*i*_ in the smart card are equal. If the values are unequal, the smart card rejects the login request. Otherwise, the legitimacy of *U*_*i*_ is ensured, and *U*_*i*_ can read the information stored in the smart card.
**Step L2.** *U*_*i*_ computes *TC*_*i*_ = *PTC*_*i*_⊕*h*(*r*_*i*_⊕*PW*_*i*_) to find its temporal credential *TC*_*i*_.

### 3.3. Authentication and key agreement phase

After ensuring the legitimacy of *U*_*i*_ and finding the temporal credential *TC*_*i*_, the system must complete mutual authentication among *U*_*i*_, the *GWN*, and *S*_*j*_. The first step of the mutual authentication phase involves identity verification for *U*_*i*_, which is conducted by the *GWN*. Afterward, the second step entails identity verification of the *GWN*, which is conducted by *S*_*j*_. The third step involves identity verification for *S*_*j*_, which is conducted by *U*_*i*_ as well as the *GWN*. Finally, a session key *KEY*_*ij*_ is negotiated between *U*_*i*_ and *S*_*j*_ to conduct encryption during data transmission later on. The steps are detailed as follows (Fig 4):

**Step V1.** *U*_*i*_ → *GWN*: {*DID*_*i*_, *q*_1_, *PKS*_*i*_, *TE*_*i*_, *P*_*i*_, *N*_*i*_}. *U*_*i*_ generates a nonce *N*_*i*_ and computes *P*_*i*_ = *h*(*ID*_*i*_∥*ID*_*GWN*_∥*TE*_*i*_), *DID*_*i*_ = *ID*_*i*_ ⊕*h*(*TC*_*i*_∥*ID*_*GWN*_∥*N*_*i*_), and *q*_1_ = *h*(*ID*_*i*_∥*TC*_*i*_∥*N*_*i*_). Afterward, *U*_*i*_ randomly chooses a secret sharing key *K*_*i*_ and computes *PKS*_*i*_ = *K*_*i*_⊕*h*(*TC*_*i*_∥*N*_*i*_). After computation, *U*_*i*_ sends the login request message *m*_*1*_ = {*DID*_*i*_, *q*_1_,*PKS*_*i*_,*TE*_*i*_, *P*_*i*_, *N*_*i*_} to the *GWN*.
**Step V2.** *GWN*→ *S*_*j*_: {*DID*_*i*_, *DID*_*GWN*_, *q*_2_, *PKS*_*GWN*_, *ID*_*GWN*_, *N*_*i*_, *N*_*GWN*_}. After obtaining message *m*_*1*_, the *GWN* computes *TC*_*i*_ = *h*(*P*_*i*_∥*K*_*GWN-U*_∥*TE*_*i*_), *ID*_*i*_ = *DID*_*i*_ ⊕*h*(*TC*_*i*_∥*ID*_*GWN*_∥*N*_*i*_), and $q_{1}^{*}$ = *h*(*ID*_*i*_∥*TC*_*i*_∥*N*_*i*_). The *GWN* then verifies whether $q_{1}^{*}$ and *q*_1_ are equal. If $q_{1}^{*}$ ≠ *q*_1_, then the *GWN* terminates the request and sends a reject message to *U*_*i*_. Otherwise, the legitimacy of *U*_*i*_ is ensured, and the *GWN* accepts the login request. The *GWN* then records the login status of *U*_*i*_ to indicate that *Ui* is logging in to the system. The *GWN* computes *K*_*i*_ = *PKS*_*i*_ ⊕*h*(*TC*_*i*_∥*N*_*i*_). At this point, the *GWN* selects a proper sensor node *S*_*j*_ with identification *SID*_*j*_ and calculates its temporal credential *TC*_*j*_ = *h*(*K*_*GWN-S*_∥*SID*_*j*_). The *GWN* then generates a nonce *N*_*GWN*_ and computes *DID*_*GWN*_ = *ID*_*i*_ ⊕*h*(*TC*_*j*_∥*DID*_*i*_∥*N*_*GWN*_), *q*_2_ = *h*(*ID*_*i*_∥*TC*_*j*_∥*N*_*GWN*_), and *PKS*_*GWN*_ = *K*_*i*_ ⊕*h*(*TC*_*j*_∥*N*_*GWN*_). After computation, the *GWN* sends the message *m*_*2*_ = {*DID*_*i*_, *DID*_*GWN*_, *q*_2_, *PKS*_*GWN*_, *ID*_*GWN*_, *N*_*i*_, *N*_*GWN*_}to *S*_*j*_.
**Step V3.** *S*_*j*_ →*U*_*i*_, *GWN*: {*SID*_*j*_, *q*_3_, *PKS*_*j*_, *N*_*i*_, *N*_*GWN*_}. After receiving message *m*_*2*_, *S*_*j*_ assesses *ID*_*GWN*_ to verify whether the *GWN* is a participant. If verification is true, *S*_*j*_ computes *ID*_*i*_ = *DID*_*GWN*_ ⊕*h*(*TC*_*j*_∥*DID*_*i*_∥*N*_*GWN*_) and $q_{2}^{*}$ = *h*(*ID*_*i*_∥*TC*_*j*_∥*N*_*GWN*_). *S*_*j*_ then verifies whether $q_{2}^{*}$ and *q*_2_ are equal. If $q_{2}^{*}$ ≠ *q*_2_, then *S*_*j*_ terminates the request and returns a reject message. Otherwise, the legitimacy of the *GWN* is ensured, and *S*_*j*_ accepts the request. *S*_*j*_ computes *K*_*i*_ = *PKS*_*GWN*_ ⊕*h*(*TC*_*j*_∥*N*_*GWN*_). Afterward, *S*_*j*_ randomly selects a secret sharing key *K*_*j*_. *S*_*j*_ computes *q*_3_ = *h*(*ID*_*i*_∥*SID*_*j*_∥*K*_*i*_∥*N*_*i*_∥*N*_*GWN*_) and *PKS*_*j*_ = *K*_*j*_⊕*h*(*K*_*i*_∥*N*_*i*_∥*N*_*GWN*_). After computation, *S*_*j*_ sends the message *m*_*3*_ = {*SID*_*j*_, *q*_3_, *PKS*_*j*_, *N*_*i*_, *N*_*GWN*_}to *U*_*i*_ and the *GWN*.
**Step V4**. After receiving the message *m*_*3*_, *U*_*i*_ and the *GWN* separately compute $q_{3}^{*}$ = *h*(*ID*_*i*_∥*SID*_*j*_∥*K*_*i*_∥*N*_*i*_∥*N*_*GWN*_). After computation, the *GWN* verifies whether $q_{3}^{*}$ and *q*_3_ are equal. If $q_{3}^{*}$ = *q*_3_, then the *GWN* can verify the legitimacy of *S*_*j*_. User *U*_*i*_ also verifies whether $q_{3}^{*}$ and *q*_3_ are equal. If $q_{3}^{*}$ = *q*_3_, then *U*_*i*_ can verify the legitimacy of *S*_*j*_ and the *GWN*. Afterward, *U*_*i*_ and the *GWN* separately compute *K*_*j*_ = *PKS*_*j*_⊕*h*(*K*_*i*_∥*N*_*i*_∥*N*_*GWN*_). Finally, after ending the mutual authentication phase, *U*_*i*_, the *GWN*, and *S*_*j*_ separately generate the shared session key *KEY*_*ij*_ by computing *KEY*_*ij*_ = *h*(*K*_*i*_∥*K*_*j*_∥*N*_*i*_∥*N*_*GWN*_∥*SID*_*j*_).

### 3.4. Password change phase

**Fig 5:**
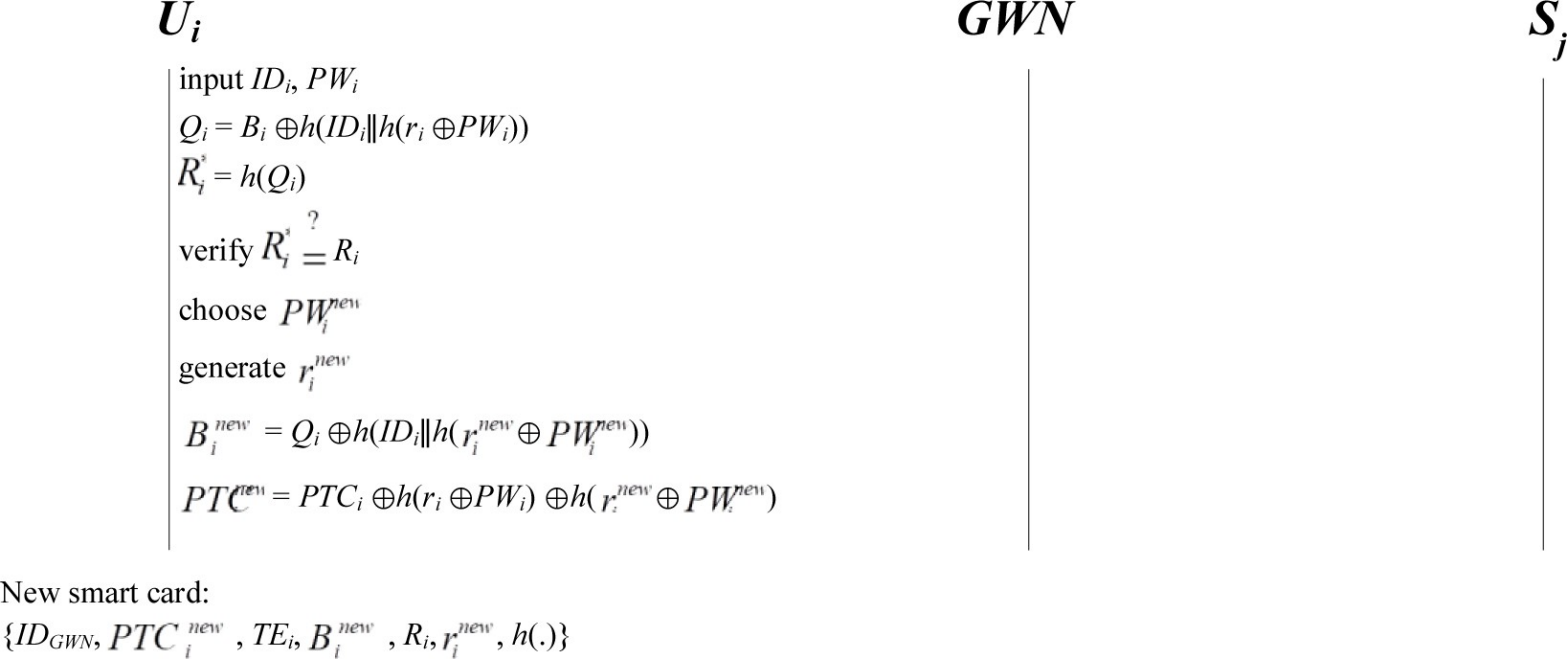
Password change phase in the proposed scheme (*U*_*i*_ can update the password without requiring the communication with the *GWN* and *S*_*j*_).

To update or change the password, a user *U*_*i*_ must insert his/her smart card into the smart card reader. Afterward, *U*_*i*_ gives *ID*_*i*_ and *PW*_*i*_, which correspond to the smart card. In the first step of the password change phase, the smart card of *U*_*i*_ computes verification information $R_{i}^{*}$ and then verifies it with the stored *R*_*i*_ in the smart card. After passing verification, the legitimacy of *U*_*i*_ is ensured. *U*_*i*_ can then read the information stored in the smart card. The second step involves finding the updated value of the parameters {$PTC_{i}^{new}$, $B_{i}^{new}$, $r_{i}^{new}$}. Finally, the smart card replaces the old value of the parameters {*PTC*_*i*_, *B*_*i*_, *r*_*i*_} in the smart card with the updated value of the parameters {$PTC_{i}^{new}$, $B_{i}^{new}$, $r_{i}^{new}$}. The steps are detailed as follows (Fig 5):

**Step P1.** A user *U*_*i*_ inserts a smart card into the smart card reader and gives (*ID*_*i*_, *PW*_*i*_). The smart card of *U*_*i*_ calculates *Q*_*i*_ = *B*_*i*_⊕*h*(*ID*_*i*_∥*h*(*r*_*i*_⊕*PW*_*i*_)) and $R_{i}^{*}$ = *h*(*Q*_*i*_) and then verifies whether $R_{i}^{*}$ and the stored *R*_*i*_ in the smart card are equal. If the values are unequal, the smart card rejects the login request. Otherwise, the legitimacy of *U*_*i*_ is ensured, and *U*_*i*_ can read the information stored in the smart card.
**Step P2.** The user *U*_*i*_ selects a new password $PW_{i}^{new}$, and then *U*_*i*_ generates a random number $r_{i}^{new}$. Then, the smart card calculates $B_{i}^{new}$ = *Q*_*i*_⊕*h*(*ID*_*i*_∥*h* ($r_{i}^{new}$⊕$PW_{i}^{new}$)), $PTC_{i}^{new}$ = *PTC*_*i*_⊕*h*(*r*_*i*_⊕*PW*_*i*_)⊕*h*($r_{i}^{new}$⊕$PW_{i}^{new}$).
**Step P3.** The parameters {*PTC*_*i*_, *B*_*i*_, *r*_*i*_} in the smart card are replaced with new parameters {$PTC_{i}^{new}$, $B_{i}^{new}$, $r_{i}^{new}$}. Finally, the smart card contains {*ID*_*GWN*_, $PTC_{i}^{new}$, *TE*_*i*_, $B_{i}^{new}$, *R*_*i*_, $r_{i}^{new}$, *h*(.)}.

## 4. Security analysis

This section presents the security analysis of the proposed scheme and proves its security strength. Our scheme can overcome the weaknesses of previous schemes. Our proposed scheme has the following main security features.

### 4.1. Mutual authentication and session key agreement

Mutual authentication is a critical feature for verifying mutual validity among the *GWN*, *U*_*i*_, and *S*_*j*_ in WSNs. Because encryption and a message authentication code (MAC) are required to protect data transmission between *U*_*i*_ and *S*_*j*_, a session key must be negotiated in advance between these two participants [7]. In this section, we first illustrate the mutual authentication analysis of the proposed scheme, then we present the formal proofs. In the authentication and key agreement phase of the proposed scheme, mutual authentication between the *GWN* and *S*_*j*_ is accomplished by calculating verification information *q*_2_ and *q*_3_. In Step V3, *S*_*j*_ can verify the legitimacy of the *GWN* after determining whether *q*_2_ and $q_{2}^{*}$ are equal, where *q*_2_ = *h*(*ID*_*i*_∥*TC*_*j*_∥*N*_*GWN*_). Temporal credential *TC*_*j*_ is included in verification information *q*_2_. This shows that the sensor node *S*_*j*_ can authenticate the validity of the *GWN*. In Step V4, the *GWN* can verify the legitimacy of *S*_*j*_ after confirming whether *q*_3_ and $q_{3}^{*}$ are equal, where *q*_3_ = *h*(*ID*_*i*_∥*SID*_*j*_∥*K*_*i*_∥*N*_*i*_∥*N*_*GWN*_). A secret sharing key *K*_*i*_ is included in verification information *q*_3_. This shows that the *GWN* can authenticate *S*_*j*_. By contrast, mutual authentication between *U*_*i*_ and the *GWN* is accomplished by calculating verification information *q*_1_ and *q*_3_. In Step V2, the *GWN* can verify the legitimacy of *U*_*i*_ after determining whether $q_{1}^{*}$ and *q*_1_ are equal, where *q*_1_ = *h*(*ID*_*i*_∥*TC*_*i*_∥*N*_*i*_). Temporal credential *TC*_*i*_ is included in verification information *q*_1_. This shows that the *GWN* can authenticate the user *U*_*i*_. In Step V4, *U*_*i*_ can verify the legitimacy of *S*_*j*_ after confirming whether *q*_3_ and $q_{3}^{*}$ are equal, where *q*_3_ = *h*(*ID*_*i*_∥*SID*_*j*_∥*K*_*i*_∥*N*_*i*_∥*N*_*GWN*_). A secret sharing key *K*_*i*_ is included in verification information *q*_3_. This shows that the user *U*_*i*_ can authenticate the sensor node *S*_*j*_. In addition, because *S*_*j*_ has authenticated the validity of the *GWN*, the user *U*_*i*_ further authenticates the validity of the *GWN* as well. Therefore, on the basis of temporal credential signing and the secret sharing key, *U*_*i*_, *S*_*j*_, and the *GWN* can mutually authenticate each other in the proposed protocol. In Step V4, after completing the mutual authentication phase, *U*_*i*_, the *GWN*, and *S*_*j*_ can separately generate the shared session key *KEY*_*ij*_ by computing *KEY*_*ij*_ = *h*(*K*_*i*_∥*K*_*j*_∥*N*_*i*_∥*N*_*GWN*_∥*SID*_*j*_), where secret sharing key *K*_*i*_ and *K*_*j*_ are selected randomly. This shows that *U*_*i*_, *S*_*j*_, and the *GWN* can share a common session key after finishing the mutual authentication phase. The common session key is validated by *U*_*i*_, the *GWN*, and *S*_*j*_. This illustration indicates that our scheme provides session key agreement and mutual authentication. The formal proofs are given in the following lemmas and Proposition 1. We use the BAN logic method [3, 21–24] to formally validate the mutual authentication and session key agreement of our scheme. The BAN logic method is widely used to validate authentication and key establishment protocols [3, 21–24]. The BAN logic method accomplishes to introduce the logic of authentication and explain the protocols step-by-step. The notations of BAN logic are presented in Table 2. In Table 2, the symbols X and Y range over statements; *Q* and *P* are principals [20–22, 42].

**Table 2:** Notations of BAN logic.

Notation	Definition
*P*◁*X*	*P* ***sees*** *X* : *P* can receive and read *X* (possibly after doing some decryption).
*P*|~*X*	*P* ***said*** *X* : *P* once said *X*. *P* once sent a message including the statement *X*.
*P*|⇒*X*	*P* ***controls*** *X* : *P* has jurisdiction over *X*.
*P*|≡*X*	*P* ***believes*** *X* : *P* is entitled to believe *X*.
#(*X*)	***fresh****(X)* : *X* is regarded as a fresh statement.
〈*X*〉_*Y*_	*X* is combined with *Y*; *Y* is a secret.
(*X*,*Y*)	*X* and *Y* are said simultaneously.
*P* $\overset{K}{↔}$ *Q*	*P* and *Q* share a common key *K*.
*P* $\underset{\leftarrow }{\underset{\to }{Y}}$ *Q*	Statement *Y* is identified only to *P* and *Q*.

The essential logical postulates for the BAN logic are listed as follows [20–22, 42]:

1. *Freshness-propagation* rule: $\frac{P|\equiv \#(X)}{P|\equiv \#(X,Y)}$. That is, if *P* is entitled to believe that one part of a formula (*X*,*Y*) is fresh, then he also is entitled to believe that the entire formula (*X*,*Y*) must also be fresh.
2. *Receiving* rule: $\frac{P⊲(X,Y)}{P⊲X}$ and $\frac{P⊲{\langle X\rangle }_{Y}}{P⊲X}$. That is, if a principal *P* can receive and read a formula (*X*,*Y*) or formula 〈*X*〉_*Y*_, then he also can receive and read its components *X*.
3. *Nonce-verification* rule: $\frac{P|\equiv \#(X),\;P|\equiv Q|∼X}{P|\equiv Q|\equiv X}$. That is, if *P* is entitled to believes that *X* is a fresh statement and that *Q* once said *X*, then *P* believes that *Q* believes *X*.
4. *Jurisdiction* rule: $\frac{P|\equiv Q|\Rightarrow X,P|\equiv Q|\equiv X}{P|\equiv X}$. That is, if *P* believes that *Q* has jurisdiction over *X* and *P* believes that *Q* believes *X*, then *P* believes *X*.
5. *Message-meaning* rule: $\frac{P|\equiv Q\begin{array}{c} \underset{\leftarrow }{\underset{\to }{Y}} \end{array}P,P⊲{\langle X\rangle }_{Y}}{P|\equiv Q|∼X}$. That is, if *P* is entitled to believe that the key *Y* is shared with *Q*, and *P* sees *X* encrypted under *Y*, then *P* is entitled to believe that *Q* once said *X*.
6. *Session-key* rule: $\frac{P|\equiv \#(K),\;P|\equiv Q|\equiv X}{P|\equiv P\overset{K}{↔}Q}$, where statement *X* is an element of the combination session key *K* [21, 44]. That is, if *P* is entitled to believe that *K* is a fresh statement and that *Q* believes *X*, then *P* believes that *P* and *Q* share a common key *K*.

To validate the proposed protocol, we first summarize our scheme in the generic form [20, 21, 42]:

Message *m*_*1*_. *U*_*i*_ → *GWN*: {*DID*_*i*_, *q*_1_, *PKS*_*i*_, *TE*_*i*_, *P*_*i*_, *N*_*i*_}

= {*ID*_*i*_⊕*h*(*TC*_*i*_∥*ID*_*GWN*_∥*N*_*i*_), *h*(*ID*_*i*_∥*TC*_*i*_∥*N*_*i*_), *K*_*i*_⊕*h*(*TC*_*i*_∥*N*_*i*_), *TE*_*i*_,

*h*(*ID*_*i*_∥*ID*_*GWN*_∥*TE*_*i*_), *N*_*i*_}.

Message *m*_*2*_. *GWN*→ *S*_*j*_: {*DID*_*i*_, *DID*_*GWN*_, *q*_2_, *PKS*_*GWN*_, *ID*_*GWN*_, *N*_*i*_, *N*_*GWN*_}

= {*ID*_*i*_ ⊕*h*(*TC*_*i*_∥*ID*_*GWN*_∥*N*_*i*_), *ID*_*i*_ ⊕*h*(*TC*_*j*_∥*DID*_*i*_∥*N*_*GWN*_),

*h*(*ID*_*i*_∥*TC*_*j*_∥*N*_*GWN*_), *K*_*i*_⊕*h*(*TC*_*j*_∥*N*_*GWN*_), *ID*_*GWN*_, *N*_*i*_, *N*_*GWN*_}.

Message *m*_*3*_. *S*_*j*_ →*GWN*: {*SID*_*j*_, *q*_3_, *PKS*_*j*_, *N*_*i*_, *N*_*GWN*_}

= {*SID*_*j*_, *h*(*ID*_*i*_∥*SID*_*j*_∥*K*_*i*_∥*N*_*i*_∥*N*_*GWN*_), *K*_*j*_⊕*h*(*K*_*i*_∥*N*_*i*_∥*N*_*GWN*_),*N*_*i*_, *N*_*GWN*_}.

Message *m*_*3*_. *S*_*j*_ →*U*_*i*_: {*SID*_*j*_, *q*_3_, *PKS*_*j*_, *N*_*i*_, *N*_*GWN*_}

= {*SID*_*j*_, *h*(*ID*_*i*_∥*SID*_*j*_∥*K*_*i*_∥*N*_*i*_∥*N*_*GWN*_), *K*_*j*_⊕*h*(*K*_*i*_∥*N*_*i*_∥*N*_*GWN*_), *N*_*i*_, *N*_*GWN*_}.

Subsequently, we transform the generic form into the idealized form:

I_1_. *U*_*i*_ → *GWN*: ${\langle N_{i}\rangle }_{TC_{i}}$, ${\langle N_{i}\rangle }_{TC_{i}}$, $\langle \langle N_{i}\rangle_{TC_{i}}\rangle_{K_{i}}$

I_2_. *GWN*→ *S*_*j*_: ${\langle N_{i}\rangle }_{TC_{i}}$, ${\langle N_{GWN}\rangle }_{TC_{j}}$, ${\langle N_{GWN}\rangle }_{TC_{j}}$, $\langle \langle N_{GWN}\rangle_{TC_{j}}\rangle_{K_{i}}$

I_3_. *S*_*j*_ → *GWN*: ${\langle N_{i},N_{GWN}\rangle }_{K_{i}}$, ${\langle {\langle N_{i},N_{GWN}\rangle }_{K_{i}}\rangle }_{K_{j}}$

I_4_. *S*_*j*_ →*U*_*i*_:${\langle N_{i},N_{GWN}\rangle }_{K_{i}}$, $\langle \langle N_{i},N_{GWN}\rangle_{K_{i}}\rangle_{K_{j}}$

To analyze our scheme, we use the following assumptions:

A_1_. $GWN|\equiv U_{i}\begin{array}{c} \underset{\leftarrow }{\underset{\to }{TC_{i}}} \end{array}GWN$               A_2_. $GWN|\equiv S_{j}\begin{array}{c} \underset{\leftarrow }{\underset{\to }{K_{i}}} \end{array}GWN$A_3_. $S_{j}|\equiv GWN\begin{array}{c} \underset{\leftarrow }{\underset{\to }{TC_{j}}} \end{array}S_{j}$                  A_4_. $U_{i}|\equiv S_{j}\begin{array}{c} \underset{\leftarrow }{\underset{\to }{K_{i}}} \end{array}U_{i}$A_5_. *GWN*|≡#(*N*_*i*_)                       A_6_. *S*_*j*_|≡#(*N*_*GWN*_)

A_7_. *U*_*i*_|≡#(*N*_*i*_,*N*_*GWN*_)              A_8_. *GWN*|≡#(*N*_*i*_,*N*_*GWN*_)

A_9_. *GWN*|≡*U*_*i*_|⇒*N*_*i*_              A_10_. *S*_*j*_|≡*GWN*|⇒*N*_*GWN*_

A_11_.*GWN*|≡*S*_*j*_|⇒(*N*_*i*_,*N*_*GWN*_)              A_12_. *U*_*i*_|≡*S*_*j*_|⇒(*N*_*i*_,*N*_*GWN*_)

**Lemma 1**. *The GWN in our scheme can authenticate U*_*i*_; *S*_*j*_ *can authenticate the GWN*.

**Proof:** In our scheme, *U*_*i*_ produces a nonce *N*_*i*_. Then, *U*_*i*_ transmits *N*_*i*_ to the *GWN*. After obtaining *N*_*i*_, the *GWN* generates a nonce *N*_*GWN*_ and then sends nonces (*N*_*i*_, *N*_*GWN*_) to *S*_*j*_.

To prove that the *GWN* can authenticate *U*_*i*_, the following belief must be demonstrated:

B_1_. *GWN*|≡*N*_*i*_

To prove that *S*_*j*_ can authenticate the *GWN*, the following belief must be demonstrated:

B_2_. *S*_*j*_|≡*N*_*GWN*_

The steps for proving B_1_:

S_1_. *GWN* ***sees*** ${\langle N_{i}\rangle }_{TC_{i}}$ (Apply the *Receiving* rule and I_1_)
S_2_. *GWN* ***believes*** *U*_*i*_ ***said*** *N*_*i*_. (Apply the *Message-meaning* rule, A_1_, and S_1_)
S_3_. *GWN* ***believes*** *U*_*i*_ ***believes*** *N*_*i*_. (Apply the *Nonce-verification* rule, A_5_, and S_2_)
S_4_. *GWN* ***believes*** *N*_*i*_. That is, *GWN*|≡*N*_*i*_ (Apply the *Jurisdiction* rule, A_9_, and S_3_)

Consequently, the *GWN* authenticates *U*_*i*_.

Similarly, the steps of the proof for B_2_:

S_5_. *S*_*j*_ ***sees*** ${\langle N_{GWN}\rangle }_{TC_{j}}$ (Apply I_2_ and *Receiving* rule)
S_6_. *S*_*j*_ ***believes*** *GWN* ***said*** *N*_*GWN*_. (Apply the *Message-meaning* rule, A_3_, and S_5_)
S_7_. *S*_*j*_ ***believes*** *GWN* ***believes*** *N*_*GWN*_. (Apply the *Nonce-verification* rule, A_6_, and S_6_)
S_8_. *S*_*j*_ ***believes*** *N*_*GWN*_. That is, *S*_*j*_|≡*N*_*GWN*_ (Apply the *Jurisdiction* rule, A_10_, and S_7_).

**Lemma 2**. *The GWN in our scheme can authenticate S*_*j*_ *; U*_*i*_ *can also authenticate S*_*j*_.

**Proof:** In our scheme, after receiving nonces (*N*_*i*_, *N*_*GWN*_), the *S*_*j*_ returns (*N*_*i*_, *N*_*GWN*_) to the *GWN* and *U*_*i*_.

To prove that the *GWN* can authenticate *S*_*j*_, the following belief must be demonstrated:

B_3_. *GWN*|≡(*N*_*i*_,*N*_*GWN*_)

To prove that the *U*_*i*_ can authenticate *S*_*j*_, the following belief must be demonstrated:

B_4_. *U*_*i*_|≡(*N*_*i*_,*N*_*GWN*_)

The steps of the proof for B_3_:

S_9_. *GWN* ***sees*** ${\langle N_{i},N_{GWN}\rangle }_{K_{i}}$ (Apply the *Receiving* rule and I_3_)
S_10_. *GWN* ***believes*** *S*_*j*_ ***said*** (*N*_*i*_,*N*_*GWN*_). (Apply the *Message-meaning* rule, A_2_, and S_9_)
S_11_. *GWN* ***believes*** *S*_*j*_ ***believes*** (*N*_*i*_,*N*_*GWN*_).(Apply the *Nonce-verification* rule, A_8_, and S_10_)
S_12_. *GWN* ***believes*** (*N*_*i*_,*N*_*GWN*_). That is, *GWN*|≡(*N*_*i*_,*N*_*GWN*_) (Apply the *Jurisdiction* rule, A_11_, and S_11_).

Consequently, the *GWN* can authenticate *S*_*j*_.

Similarly, the steps of the proof for B_4_:

S_13_. *U*_*i*_ ***sees*** ${\langle N_{i},N_{GWN}\rangle }_{K_{i}}$ (Apply the *Receiving* rule and I_4_)
S_14_. *U*_*i*_ ***believes*** *S*_*j*_ ***said*** (*N*_*i*_,*N*_*GWN*_). (Apply the *Message-meaning* rule, A_4_, and S_13_)
S_15_. *U*_*i*_ ***believes*** *S*_*j*_ ***believes*** (*N*_*i*_,*N*_*GWN*_). (Apply the *Nonce-verification* rule, A_7_, and S_14_)
S_16_. *U*_*i*_ ***believes*** (*N*_*i*_,*N*_*GWN*_). That is, *U*_*i*_|≡(*N*_*i*_,*N*_*GWN*_). (Apply the *Jurisdiction* rule, A_12_, and S_15_)

**Lemma 3.** *In our scheme*, *the GWN*, *U*_*i*_, *and S*_*j*_ *can coordinate the common session key KEY*_*ij*_.

**Proof:** To prove that *U*_*i*_, the *GWN*, and *S*_*j*_ in our scheme can share a session key *KEY*_*ij*_ = *h*(*K*_*i*_∥*K*_*j*_∥*N*_*i*_∥*N*_*GWN*_∥*SID*_*j*_), the following beliefs must be demonstrated:

B_5_. $U_{i}|\equiv U_{i}\overset{KEY_{ij}}{↔}GWN$

B_6_. $GWN|\equiv GWN\overset{KEY_{ij}}{↔}U_{i}$

B_7_. $S_{j}|\equiv S_{j}\overset{KEY_{ij}}{↔}GWN$

B_8_. $GWN|\equiv GWN\overset{KEY_{ij}}{↔}S_{j}$ The steps for proving B_5_ are:

S_17_. *U*_*i*_ ***believes*** *S*_*j*_ ***believes*** (*N*_*i*_,*N*_*GWN*_). (Apply S_15_)
S_18_. *S*_*j*_ ***believes*** *GWN* ***believes*** *N*_*GWN*_. (Apply S_7_)
S_19_. *U*_*i*_ ***believes*** *GWN* ***believes*** *N*_*GWN*_. (Apply the Lemma 1, the Lemma 2, S_17_, and S_18_)
S_20_. *U*_*i*_ ***believes fresh*** (*N*_*i*_, *N*_*GWN*_). (Apply A_7_)
S_21_. *U*_*i*_ ***believes fresh*** (*KEY*_*ij*_). (Apply S_20_ and *Freshness-propagation* rule)
S_22_. *Ui* ***believes*** $U_{i}\overset{KEY_{ij}}{↔}GWN$. That is, $U_{i}|\equiv U_{i}\overset{KEY_{ij}}{↔}GWN$. (Apply the *Session-key* rule, S_19_, and S_21_)

Consequently, *U*_*i*_ believes that *U*_*i*_ shares the session key *KEY*_*ij*_ with the *GWN*.

Similarly, the steps of the proof for B_6_:

S_23_. *GWN* ***believes*** *U*_*i*_ ***believes*** *N*_*i*_. (Apply S_3_)
S_24_. *GWN* ***believes fresh*** (*N*_*i*_). (Apply A_5_)
S_25_. *GWN* ***believes fresh*** (*KEY*_*ij*_). (Apply S_24_ and *Freshness-propagation* rule)
S_26_. *GWN* ***believes*** $GWN\overset{KEY_{ij}}{↔}U_{i}$. That is, $GWN|\equiv GWN\overset{KEY_{ij}}{↔}U_{i}$. (Apply S_23_, S_25_, and *Session-key* rule)

Consequently, the *GWN* believes that *GWN* shares the session key *KEY*_*ij*_ with *U*_*i*_.

The steps of the proof for B_7_ are:

S_27_. *S*_*j*_ ***believes*** *GWN* ***believes*** *N*_*GWN*_. (Apply S_7_)
S_28_. *S*_*j*_ ***believes fresh*** (*N*_*GWN*_). (Apply A_6_)
S_29_. *S*_*j*_ ***believes fresh*** (*KEY*_*ij*_). (Apply the *Freshness-propagation* rule and S_28_)
S_30_. *S*_*j*_ ***believes*** $S_{j}\overset{KEY_{ij}}{↔}GWN$. That is, $S_{j}|\equiv S_{j}\overset{KEY_{ij}}{↔}GWN$. (Apply the *Session-key* rule, S_27_, S_29_)

Consequently, *S*_*j*_ believes that *S*_*j*_ shares the session key *KEY*_*ij*_ with the *GWN*.

Similarly, the steps of the proof for B_8_ are:

S_31_. *GWN* ***believes*** *S*_*j*_ ***believes*** (*N*_*i*_,*N*_*GWN*_). (Apply S_11_)
S_32_. *GWN* ***believes fresh*** (*N*_*i*_). (Apply A_5_)
S_33_. *GWN* ***believes fresh*** (*KEY*_*ij*_). (Apply S_32_ and *Freshness-propagation* rule)
S_34_. *GWN* ***believes*** $GWN\overset{KEY_{ij}}{↔}S_{j}$. That is, $GWN|\equiv GWN\overset{KEY_{ij}}{↔}S_{j}$. (Apply S_31_, S_33_, and *Session-key* rule)

Consequently, the *GWN* believes that *GWN* shares the session key *KEY*_*ij*_ with *S*_*j*_.

**Proposition 1**. *U*_*i*_, *the GWN*, *and S*_*j*_ *in our scheme can mutually authenticate each other; they can share a common session key*.

**Proof:** From Lemma 2, *U*_*i*_ in our scheme can authenticate *S*_*j*_. In addition, *S*_*j*_ can authenticate the *GWN* (Lemma 1). Thus, *U*_*i*_ can further authenticate the *GWN* as well. Conversely, the *GWN* can authenticate *U*_*i*_ (Lemma 1). Consequently, the *GWN* and *U*_*i*_ in our scheme can mutually authenticate each other. The *GWN* can authenticate *S*_*j*_ (Lemma 2). Conversely, *S*_*j*_ can authenticate the *GWN* (Lemma 1). Consequently, the *GWN* and *S*_*j*_ in our scheme can mutually authenticate each other. Mutual authentication can be provided in our scheme. After finishing the mutual authentication, *U*_*i*_, the *GWN*, *and S*_*j*_ can share a session key *KEY*_*ij*_ = *h*(*K*_*i*_∥*K*_*j*_∥*N*_*i*_∥*N*_*GWN*_∥*SID*_*j*_) (Lemma 3). Session key agreement can also be provided in our scheme.

### 4.2. Password protection, guessing attack resistance, and stolen smart card attack resistance

When a user’s smart card is stolen or lost in a stolen smart card attack, an adversary can acquire information from the smart card. Then, the adversary masquerades as an authorized user to access to the GWN. However, password protection functionality can prevent the leakage of password information, such that the adversary cannot obtain useful information to perform an off-line password guessing attack.

**Proposition 2**. *The proposed scheme can provide password protection*, *guessing attack resistance*, *and stolen smart card attack resistance*.

**Proof:** In our scheme, the password presents with the *h*(*r*_*i*_ ⊕*PW*_*i*_) form, in which *PW*_*i*_ and *r*_*i*_ are hidden. *h*(*r*_*i*_⊕*PW*_*i*_) is not stored in the smart card, the *GWN*, or any other device. Thus, the adversary cannot directly obtain *PW*_*i*_ by performing an off-line password guessing attack on *h*(*r*_*i*_⊕*PW*_*i*_) [45]. Therefore, the proposed scheme can provide password protection and guessing attack resistance. Moreover, smart card secrets can be breached by monitoring power consumption or by analyzing leaked information [25, 42, 46]. When the adversary has a smart card that has been lost by its legitimate owner, the adversary can acquire the secret parameters from that smart card by applying the previously discussed method. We can prove that the proposed scheme can also provide stolen smart card attack resistance. That is, in the proposed scheme, the adversary cannot masquerade as a legitimate user to log in to the *GWN* when the adversary has obtained a legitimate user's smart card. Suppose that when the smart card of user *U*_*i*_ is stolen or lost, the adversary obtains that the smart card. The adversary can obtain the secret parameters {*ID*_*GWN*_, *PTC*_*i*_, *TE*_*i*_, *B*_*i*_, *R*_*i*_, *r*_*i*_, *h*(.)} from the smart card. To impersonate a legitimate user, the adversary must produce a new $N_{i}^{"}$, randomly choose an imitative secret sharing key $K_{i}^{"}$, and create an imitative login request message {$DID_{i}^{"}$, $q_{1}^{"}$, $PKS_{i}^{"}$, *TE*_*i*_, *P*_*i*_, $N_{i}^{"}$} for the *GWN*. The imitative parameters {$DID_{i}^{"}$, $q_{1}^{"}$, $PKS_{i}^{"}$, *P*_*i*_} are obtained using the following equations:

$DID_{i}^{"}$ = ID_i_ ⊕h(TC_i_∥ID_GWN_∥$N_{i}^{"}$),

$q_{1}^{"}$ = h(ID_i_∥TC_i_∥$N_{i}^{"}$),

$PKS_{i}^{"}$ = $K_{i}^{"}$⊕h(TC_i_∥$N_{i}^{"}$),

P_i_ = h(ID_i_∥ID_GWN_∥TE_i_).

Therefore, to obtain the imitative parameters {$DID_{i}^{"}$, $q_{1}^{"}$, $PKS_{i}^{"}$, *P*_*i*_}, the adversary must first obtain *TC*_*i*_ and *ID*_*i*_ by using the following equations:

TC_i_ = h(P_i_∥K_GWN-U_∥TE_i_),

*TC*_*i*_ *= PTC*_*i*_⊕*h(r*_*i*_⊕*PW*_*i*_*)*,

*ID*_*i*_ *= DID*_*i*_⊕*h(TC*_*i*_*∥ID*_*GWN*_*∥N*_*i*_*)*.

Nevertheless, the adversary cannot acquire *TC*_*i*_ and *ID*_*i*_ because he/she does not possess *K*_*GWN-U*_ and *PW*_*i*_. Only the *GWN* knows the private key *K*_*GWN-U*_ in our scheme. As previously discussed, the proposed scheme can provide password protection, and that the adversary cannot acquire *PW*_*i*_ by executing an off-line password guessing attack. Therefore, the imitative parameter set {$DID_{i}^{"}$, $q_{1}^{"}$, $PKS_{i}^{"}$, *P*_*i*_}of a login request message is not acquired. The adversary cannot masquerade as an authorized user by only using a smart card.

### 4.3. Two-factor security

By involving a smart card and a password in the login phase, two-factor security in our scheme can be achieved [9, 37, 47, 48].

**Proposition 3**. Two-factor security can be provided in our scheme.

**Proof:** First, assume that the adversary only has the smart card of U_i_. Let us even assume that the adversary can intercept login request message *m*_1_ = {*DID*_*i*_, *q*_1_, *PKS*_*i*_, *TE*_*i*_, *P*_*i*_, *N*_*i*_}. As mentioned in Proposition 2, the adversary can obtain the secret parameters {*ID*_*GWN*_, *PTC*_*i*_, *TE*_*i*_, *B*_*i*_, *R*_*i*_, *r*_*i*_, *h*(.)} from the smart card. To impersonate a legitimate user, the adversary must produce a new $N_{i}^{"}$, randomly choose a new sharing key $K_{i}^{"}$, and create an imitative login request message {$DID_{i}^{"}$, $q_{1}^{"}$, $PKS_{i}^{"}$, TE_i_, P_i_, $N_{i}^{"}$} for the GWN, where $DID_{i}^{"}$ = ID_i_ ⊕h(TC_i_∥ID_GWN_∥$N_{i}^{"}$), $q_{1}^{"}$ = h(ID_i_∥TC_i_∥$N_{i}^{"}$), and $PKS_{i}^{"}$ = $K_{i}^{"}$⊕h(TC_i_∥$N_{i}^{"}$). Consequently, to gain the parameter set {$DID_{i}^{"}$, $q_{1}^{"}$, $PKS_{i}^{"}$}, the adversary must acquire TC_i_ and ID_i_ by applying the following equations: TC_i_ = h(P_i_∥K_GWN-U_∥TE_i_), TC_i_ = PTC_i_ ⊕h(r_i_⊕PW_i_), and ID_i_ = DID_i_⊕h(TC_i_∥ID_GWN_∥N_i_). Nevertheless, the adversary cannot acquire TC_i_ and ID_i_ because he/she does not possess K_GWN-U_ and PW_i_. Only the GWN knows the private key K_GWN-U_ in our scheme, and we have proven that the proposed scheme can provide password protection to prevent the leakage of PW_i_ information (Section 4.2). Therefore, the parameter set {$DID_{i}^{"}$, $q_{1}^{"}$, $PKS_{i}^{"}$} of the login request message is not acquired, and the adversary cannot disguise as an authorized user by only using the smart card. Secondly, assume that the adversary only has the password PW_i_ and identification ID_i_ of U_i_. Under this condition, the adversary also cannot acquire TC_i_ to calculate the parameters {$DID_{i}^{"}$, $q_{1}^{"}$, $PKS_{i}^{"}$} because he/she does not know K_GWN-U_ and PTC_i_ (which are not stored in the smart card). Therefore, the adversary cannot impersonate an authorized user when he/she either acquires information from the smart card or knows {ID_i_, PW_i_}. Our scheme can withstand this type of masquerade attack and provide two-factor security.

### 4.4. Masquerade attack resistance and replay attack resistance

Protection against masquerade attacks is a principal security feature for any remote user authentication scheme. Replay attack resistance means that the adversary cannot attempt to replay any previously intercepted message to spoof the *GWN*.

**Proposition 4**. *Our scheme can provide masquerade attack resistance and replay attack resistance*.

**Proof:** Proposition 3 has demonstrated that our scheme can protect against masquerade attacks caused by either the loss of a smart card or the revelation of sensitive identification and password details {*ID*_*i*_, *PW*_*i*_}. The reliability of our scheme against other masquerade attacks must be demonstrated. We can even assume that the adversary is a legitimate user *L* and undertakes to impersonate a user *U*_*i*_. Adversary *L* may intercept the login request message *m*_*1*_ = {*DID*_*i*_, *q*_1_, *PKS*_*i*_, *TE*_*i*_, *P*_*i*_, *N*_*i*_}. Adversary *L* can have {*ID*_*l*_, *PW*_*l*_} and acquire {*ID*_*GWN*_, *PTC*_*l*_, *TE*_*l*_, *B*_*l*_, *R*_*l*_, *r*_*l*_, *h*(.)} from his/her smart card because he/she is an admissible user. Adversary *L* generates a new nonce $N_{i}^{"}$, randomly chooses an imitative secret sharing key $K_{i}^{"}$, and creates an imitative login request message {$DID_{i}^{"}$, $q_{1}^{"}$, $PKS_{i}^{"}$, *TE*_*i*_, *P*_*i*_, $N_{i}^{"}$} for the *GWN*, where $DID_{i}^{"}$ = *ID*_*i*_ ⊕*h*(*TC*_*i*_∥*ID*_*GWN*_∥$N_{i}^{"}$), $q_{1}^{"}$ = *h*(*ID*_*i*_∥*TC*_*i*_∥$N_{i}^{"}$), and $PKS_{i}^{"}$ = $K_{i}^{"}$ ⊕*h*(*TC*_*i*_∥$N_{i}^{"}$). Nevertheless, adversary *L* still cannot acquire *TC*_*i*_ and *ID*_*i*_ to calculate the parameters {$DID_{i}^{"}$, $q_{1}^{"}$, $PKS_{i}^{"}$} because he/she does not possess *K*_*GWN-U*_ and *PW*_*i*_ (Proposition 2). In addition, adversary *L* cannot compute the shared session key *KEY*_*ij*_ = *h*(*K*_*i*_∥*K*_*j*_∥*N*_*i*_∥*N*_*GWN*_∥*SID*_*j*_) because he or she does not know *K*_*i*_ and *K*_*j*_ in *KEY*_*ij*_. Thus, adversary *L* cannot impersonate any other legitimate user. Consequently, our scheme can protect against masquerade attacks when an adversary impersonates any other legitimate user. Adversary *L* can undertake to replay the intercepted message {*DID*_*i*_, *q*_1_, *PKS*_*i*_, *TE*_*i*_, *P*_*i*_, *N*_*i*_} to the *GWN*. However, after receiving message *m*_*3*_ = {*SID*_*j*_, *q*_3_, *PKS*_*j*_, *N*_*i*_, *N*_*GWN*_}, adversary *L* cannot compute the shared session key *KEY*_*ij*_ = *h*(*K*_*i*_∥*K*_*j*_∥*N*_*i*_∥*N*_*GWN*_∥*SID*_*j*_) because he or she cannot obtain *K*_*i*_ and *K*_*j*_ in *KEY*_*ij*_. Consequently, resistance to replay attacks is guaranteed as well. Next, we prove that an adversary cannot masquerade as a sensor node to spoof the user. Suppose adversary *L* has intercepted message *m*_*2*_ when the *GWN* attempts to send it to *S*_*j*_; that is, the message {*DID*_*i*_, *DID*_*GWN*_, *q*_2_, *PKS*_*GWN*_, *ID*_*GWN*_, *N*_*i*_, *N*_*GWN*_}. To masquerade as a sensor node to spoof the user, the adversary must randomly choose an imitative secret sharing key $K_{j}^{"}$ and send an imitative response message {*SID*_*j*_, *q*_3_, $PKS_{j}^{"}$, *N*_*i*_, *N*_*GWN*_} to the *GWN*, where *q*_3_ = *h*(*ID*_*i*_∥*SID*_*j*_∥*K*_*i*_∥*N*_*i*_∥*N*_*GWN*_) and $PKS_{j}^{"}$ = $K_{j}^{"}$⊕*h*(*K*_*i*_∥*N*_*i*_∥*N*_*GWN*_). To obtain the parameters {*q*_3_, $PKS_{j}^{"}$}, the adversary must first know *K*_*i*_. Moreover, *K*_*i*_ can be obtained by using the equation *K*_*i*_ = *PKS*_*GWN*_⊕*h*(*TC*_*j*_∥*N*_*GWN*_). Nevertheless, the adversary cannot acquire *K*_*i*_ because he/she does not possess the temporal credential *TC*_*j*_. Therefore, the parameters {*q*_3_, $PKS_{j}^{"}$} cannot be acquired, and the adversary cannot send an imitative response message {*SID*_*j*_, *q*_3_, $PKS_{j}^{"}$, *N*_*i*_, *N*_*GWN*_} to the *GWN*. Consequently, our scheme can protect against masquerade attacks when an adversary masquerades as a sensor node to spoof the user.

### 4.5. Stolen verifier attack resistance and insider attack resistance

The stolen verifier attack means that the adversary steals the verification table from the *GWN* or *S*_*j*_. By contrast, an insider attack involves any privileged insider of the *GWN* purposely obtaining a user password, which leads to security defects in the remote user authentication scheme [41, 49].

**Proposition 5**. *Our scheme can protect against stolen verifier attacks and insider attacks*.

**Proof:** The *GWN* and *S*_*j*_ in our scheme do not retain any verification table for verifying the legitimacy of registered users or sensor nodes. Therefore, the adversary cannot find any verifiable information in the *GWN* or *S*_*j*_ to impersonate a legitimate user. Consequently, our scheme can protect against stolen verifier attacks [9, 40]. Moreover, because *U*_*i*_ presents *h*(*r*_*i*_⊕*PW*_*i*_) to register with the *GWN*. *r*_*i*_ and *PW*_*i*_ are hidden from the *GWN*. In addition, the *GWN* does not store any verifier *h*(*r*_*i*_⊕*PW*_*i*_). The privileged insider of the *GWN* cannot acquire *PW*_*i*_ by executing any off-line password guessing attack [45]. Consequently, our scheme can resist insider attacks [41].

### 4.6. Password updating, adding new user functionality, and time synchronization avoidance

In our scheme, users are not obliged to share their IDs and passwords with the *GWN* before the registration. During the registration process, a new user *U*_*i*_ can freely choose some identification string *ID*_*i*_ and password *PW*_*i*_ as favorite strings without requiring assistance from the *GWN*. Any new legitimate user can be freely added to the system after the registration. Therefore, the proposed scheme provides a convenient functionality for adding new users. Moreover, as mentioned, it is strongly recommended that for security policy, users update or change their passwords frequently to protect against compromise [32]. In the password change phase of our scheme, a legitimate user *U*_*i*_ can freely choose his/her new password to update or change the password without requiring extra communication message overhead to exchange messages with the *GWN* (Fig 5). Consequently, our scheme provides the functionalities of freely chosen passwords and efficient password updating. Finally, our scheme does not require any timestamp to verify mutual authentication among *U*_*i*_, the *GWN*, and *S*_*j*_ because our scheme is a nonce-based scheme. Consequently, our scheme is not obliged to provide synchronized time clocks for all devices [3, 17, 18], and it can avoid the time-synchronization problem for WSNs in IoT environments [3, 17, 25].

### 4.7. User anonymity (identity protection)

The user anonymity (identity protection) means that the identity of any user is disclosed only to service providers [9].

**Proposition 6**. *Our scheme can provide user anonymity to protect user identity*.

**Proof:** The adversary can intercept message *m*_*1*_ = {*DID*_*i*_, *q*_1_, *PKS*_*i*_, *TE*_*i*_, *P*_*i*_, *N*_*i*_} to acquire the identification string of *U*_*i*_,. The parameters *DID*_*i*_, *q*_1_, *PKS*_*i*_, and *P*_*i*_ are obtained using the following equations:

*DID*_*i*_ *= ID*_*i*_ ⊕*h(TC*_*i*_*∥ID*_*GWN*_*∥N*_*i*_*)*,

*q*_1_ *= h(ID*_*i*_*∥TC*_*i*_*∥N*_*i*_*)*,

*PKS*_*i*_ *= K*_*i*_⊕*h(TC*_*i*_*∥N*_*i*_*)*,

*P*_*i*_ *= h(ID*_*i*_*∥ID*_*GWN*_*∥TE*_*i*_*)*.

However, in Proposition 2, we show that the adversary cannot obtain *ID*_*i*_ and *TC*_*i*_ because he or she does not know *K*_*GWN-U*_ and *PW*_*i*_. The identification string *ID*_*i*_ also cannot be derived from the equations above. Therefore, an adversary cannot acquire *ID*_*i*_ to identify the user *U*_*i*_, and our scheme can provide user anonymity to protect user identity.

### 4.8. GWN bypassing attack resistance and GWN spoofing attack resistance

A *GWN* bypassing attack occurs when an adversary can bypass the *GWN* to forge a verification message straight to the sensor node *S*_*j*_ without passing the *GWN* login [7]. By contrast, a *GWN* spoofing attack occurs when an adversary may impersonate the *GWN* to obtain private login information of *U*_*i*_.

**Proposition 7**. *Our scheme can protect against GWN bypassing attacks and GWN spoofing attacks*.

**Proof:** To bypass the *GWN*, an adversary must send an imitative verification message *m*_*2*_ = {*DID*_*i*_, *DID*_*GWN*_, *q*_2_, *PKS*_*GWN*_, *ID*_*GWN*_, *N*_*i*_, *N*_*GWN*_} straight to *S*_*j*_, where *q*_2_ = *h*(*ID*_*i*_∥*TC*_*j*_∥*N*_*GWN*_). However, the adversary cannot obtain *q*_2_ to create an imitative message *m*_*2*_ because he or she does not know the temporal credential *TC*_*j*_; thus, the adversary cannot bypass the *GWN* to forge *m*_*2*_ to *S*_*j*_. Without *m*_*2*_, *S*_*j*_ cannot respond with any other messages. Consequently, our scheme can prevent *GWN* bypassing attacks. By contrast, the adversary may attempt to impersonate the *GWN* to acquire the secret login information of *U*_*i*_. To pose as the *GWN*, the adversary can intercept some login request message *m*_*1*_ = {*DID*_*i*_, *q*_1_, *PKS*_*i*_, *TE*_*i*_, *P*_*i*_, *N*_*i*_} and respond with an imitative message *m*_*3*_ = {*SID*_*j*_, *q*_3_, *PKS*_*j*_, *N*_*i*_, *N*_*GWN*_} to *U*_*i*_, where *q*_3_ = *h*(*ID*_*i*_∥*SID*_*j*_∥*K*_*i*_∥*N*_*i*_∥*N*_*GWN*_). Verification information *q*_3_ includes a secret sharing key *K*_*i*_. However, as mentioned in Proposition 4, the adversary cannot acquire *K*_*i*_ because he/she does not know temporal credential *TC*_*j*_. Therefore, the adversary cannot obtain *q*_3_; thus, the adversary cannot send an imitative message *m*_*3*_ = {*SID*_*j*_, *q*_3_, *PKS*_*j*_, *N*_*i*_, *N*_*GWN*_} to respond to *U*_*i*_. The adversary cannot convince *U*_*i*_ that he/she is a legitimate *GWN*. Consequently, our scheme can protect against *GWN* spoofing attacks.

## 5. Performance evaluation and functionality comparison

Performance and functionality evaluations are critical to establish validity for practical deployment. In this section, the performance and functionality of our scheme are evaluated. The performance efficiency and functional effectiveness of our authentication scheme are demonstrated.

### 5.1. Functionality comparison

Table 3 presents a functionality comparison of our scheme versus previous related schemes. In Table 3, *Yes* denotes the scheme has a security feature; *No* denotes the contrary. The weaknesses of the previous related schemes for WSNs are mentioned in Section 2 and summarized in Table 3. We present a practical scenario to show that the proposed scheme can provide secure functionality and effectiveness for WSNs in IoT environments. Suppose that an adversary, *Eve*, undertakes to damage our scheme by executing the following attacks: guessing attack, stolen smart card attack, masquerade attack, replay attack, stolen verifier attack, insider attack, user anonymity attack, or GWN bypassing attack. Section 4 has shown that our scheme has the following abilities. *Eve* cannot directly obtain a user’s password by executing a password guessing attack. When *Eve* steals a user’s smart card, she cannot impersonate an authorized user to access the system. When *Eve* is even a legitimate user who pretends to be a different legitimate user, our scheme can protect against this masquerade attack. Moreover, *Eve* may undertake to replay some intercepted message to the *GWN*. Our scheme can provide resistance to replay attacks. *Eve* cannot breach the system by stealing the verification table. Even as an insider, *Eve* cannot acquire a password by executing any password guessing attack. *Eve* may intercept a login request message from the user to acquire the identification information, but the identification information of a user cannot be derived. Finally, *Eve* cannot forge an imitative message and send it straight to the sensor node to bypass the GWN. Moreover, our scheme has other security functionalities, which include updating passwords, choosing passwords freely, adding new users, and time synchronization avoidance. Our scheme provides a secure common session key and mutual authentication. Our scheme can thus protect against all listed attacks from *Eve*.

**Table 3:** Functionality comparison of our scheme with other related schemes.

	Ours Ostad-Sharif Amin et al. Chang et al. Xue et al. Yeh et al. Khan et al. Chen et al. Das (2019)[2] (2018)[34] (2016)[35] (2013)[7] (2011)[8] (2010)[32] (2010)[33] (2009)[5]
Password protection	Yes Yes Yes No No Yes Yes No No
Stolen smart card attack resistance	Yes Yes Yes No No No No No No
Masquerade attack resistance	Yes Yes Yes No Yes Yes Yes No No
Replay attacks resistance	Yes Yes Yes No Yes No Yes Yes Yes
Insider attack resistance	Yes Yes Yes Yes No Yes Yes No No
Password updating/changing	Yes No Yes Yes No No Yes No No
Time synchronization avoidance	Yes No No No No Yes No No No
Mutual authentication	Yes No Yes Yes Yes Yes Yes Yes No
Session key agreement	Yes Yes Yes Yes Yes Yes No No No
User anonymity	Yes Yes Yes No Yes No Yes Yes Yes
GWN bypassing attack resistance	Yes Yes Yes Yes Yes Yes No No No

### 5.2. Performance evaluation

The proposed scheme comprises four phases: registration phase, login phase, authentication and key agreement phase, and password change phase. In a WSN environment, the performance of the authentication scheme is affected mainly by the authentication and key agreement phase [2, 7, 34, 35]. This phase is the main part of the authentication scheme and is what chiefly distinguishes it from the various authentication schemes in WSNs [2, 7, 34, 35]. Therefore, we focus our discussion on the performance comparison of the authentication and key agreement phase in the authentication schemes. The performance comparison is usually separated into communication costs and computational costs [2, 7, 34, 35, 42]. The computational costs are defined as the time spent by the user and service provider in the process [2, 7, 34, 35, 42]. By contrast, the communication costs are defined as the number of messages dispatched by the user and service provider in the process [9, 42]. The performance comparison of our scheme and previous related schemes is shown in Table 4. Table 4 presents the computational costs and communication costs of the authentication and key agreement phase in each authentication scheme run without the consideration of interference and packet loss [2, 7, 21, 34, 35]. The notation *T*_h_ is defined as the time complexity of the hash function; *T*_ecc_ is the time complexity of the encryption/decryption operation in elliptic curve cryptography (ECC) algorithm [7]. The computational costs of the exclusive-or operation are usually neglected because it necessitates minimal computations [2, 7, 34, 35]. We first analyze the computational costs of the authentication and key agreement phase for each scheme as follows:

1. In the authentication phase of the Ostad-Sharif et al. scheme [2], the user requires 10*T*_h_ to compute the parameters of the login request message and the response message. The GWN must spend 14*T*_h_ to compute the parameters in a response message for the user and a request message for the sensor node. The sensor node must expend 3*T*_h_ to confirm whether the verification equations hold. In addition, the user, GWN, and sensor node must expend 2*T*_h_, 3*T*_h_, and 2*T*_h_ separately to negotiate the shared session key in the key agreement phase. Accordingly, the total computational costs for the user, GWN, and sensor node are 12*T*_h_, 17*T*_h_, and 5*T*_h_, respectively [2].
2. In the authentication phase of the Amin et al. scheme [34], the user requires 13*T*_h_ to compute the parameters of the login request message and the response message. The GWN must spend 14*T*_h_ to compute the parameters in a request message for the sensor node and a response message for the user. The sensor node must expend 2*T*_h_ to confirm whether the verification equations hold. In addition, the user, GWN, and sensor node must expend 1*T*_h_, 3*T*_h_, and 2*T*_h_ separately to negotiate the shared session key in the key agreement phase. Accordingly, the total computational costs for the user, GWN, and sensor node are 14*T*_h_, 17*T*_h_, and 4*T*_h_, respectively [34].
3. In the authentication phase of the Chang et al. scheme [35], the user requires 3*T*_h_ to compute the parameters of the login request message. The sensor node must expend 1*T*_h_ to compute the parameters in a message for the GWN. The GWN must spend 5*T*_h_ to verify the login request. In addition, the user, GWN, and sensor node must expend 3*T*_h_, 3*T*_h_, and 4*T*_h_ separately to negotiate the shared session key in the key agreement phase. Accordingly, the total computational costs for the user, GWN, and sensor node are 6*T*_h_, 8*T*_h_, and 5*T*_h_, respectively [35].
4. In the authentication phase of the Xue et al. scheme [7], the user requires 5*T*_h_ to compute the parameters of the login request message. The GWN must spend 11*T*_h_ to verify the login request message and compute the parameters of the request message for the sensor node. The sensor node must expend 3*T*_h_ to confirm whether the verification equations hold. Moreover, the user, GWN, and sensor node must expend 3*T*_h_, 3*T*_h_, and 3*T*_h_ separately to negotiate the shared session key in the key agreement phase. Accordingly, the total computational costs for the user, GWN, and sensor node are 8*T*_h_, 14*T*_h_, and 6*T*_h_, respectively [7].
5. In the Khan et al. scheme [32], the user must expend 3*T*_h_ to generate a login request message. The GWN must expend 5*T*_h_ to confirm whether the verification equations hold and to calculate the parameters of the request message for the sensor node. The sensor node requires 2*T*_h_ to confirm whether the verification equations hold and to generate a response message for the GWN. However, the Khan et al. scheme does not provide the key agreement phase for the session key agreement.
6. In the Chen et al. scheme [33], the user must expend 4*T*_h_ to produce a login request message and to validate a response message. The GWN requires 5*T*_h_ to validate a login request message and to respond to a user’s request. The sensor node must expend 2*T*_h_ to verify the request message from the GWN and to generate a response message for the user. However, the Chen et al. scheme also does not provide any key agreement phase.
7. In the Das scheme [5], the user requires 3*T*_h_ to generate the login request message. The GWN must expend 4*T*_h_ to confirm whether the verification equations hold and to calculate the parameters of the request message for the sensor node. The sensor node requires 1*T*_h_ to confirm whether the verification equations hold and to generate a response message for the user. The Das scheme [5] does not provide the key agreement phase as well.
8. The Yeh et al. scheme [8] uses elliptic curve cryptography (ECC) to provide both the authentication phase and session key agreement phase. That scheme requires that the user, GWN, and sensor node expend 2*T*_ecc_ + 1*T*_h_, 4*T*_ecc_ + 3*T*_h_, and 2*T*_ecc_ + 2*T*_h_ separately to complete the authentication phase [7]. Moreover, the user, GWN, and sensor node must expend 1*T*_h_, 1*T*_h_, and 1*T*_h_ separately to compute a shared session key in the key agreement phase [7]. Accordingly, the total computational costs of the user, GWN, and sensor node are 2*T*_ecc_ + 2*T*_h_, 4*T*_ecc_ + 4*T*_h_, and 2*T*_ecc_ + 3*T*_h_, respectively [7].
9. Our proposed scheme provides both the authentication phase and key agreement phase. In the authentication phase of our scheme, the user requires only 4*T*_h_ to calculate the parameters of a login request message. The GWN expends only 8*T*_h_ to verify the login request and to calculate the parameters of the request message for the sensor node. The sensor node requires only 3*T*_h_ to confirm whether the verification equations hold. In the key agreement phase, the user, GWN, and sensor node expend only 3*T*_h_, 3*T*_h_, and 3*T*_h_, respectively, to negotiate the shared session key. Accordingly, the total computational costs for the user, GWN, and sensor node are 7*T*_h_, 11*T*_h_, and 6*T*_h_, respectively.

**Table 4:** Performance comparison of our scheme with other related schemes.

	Ours Ostad-Sharif Amin et al. Chang et al. Xue et al. Yeh et al. Khan et al. Chen et al. Das (2019)[2] (2018)[34] (2016)[35] (2013)[7] (2011)[8] (2010)[32] (2010)[33] (2009)[5]
【*Computational cost*】	
*authentication phase*	
User	4*T*_h_ 10*T*_h_ 13*T*_h_ 3*T*_h_ 5*T*_h_ 2*T*_ecc_*+*1*T*_h_ 3*T*_h_ 4*T*_h_ 3*T*_h_
GWN	8*T*_h_ 14*T*_h_ 14*T*_h_ 5*T*_h_ 11*T*_h_ 4*T*_ecc_*+*3*T*_h_ 5*T*_h_ 5*T*_h_ 4*T*_h_
Sensor node	3*T*_h_ 3*T*_h_ 2*T*_h_ 1*T*_h_ 3*T*_h_ 2*T*_ecc_*+*2*T*_h_ 2*T*_h_ 2*T*_h_ 1*T*_h_
*key agreement phase*	
User	3*T*_h_ 2*T*_h_ 1*T*_h_ 3*T*_h_ 3*T*_h_ 1*T*_h_ − * − * − *
GWN	3*T*_h_ 3*T*_h_ 3*T*_h_ 3*T*_h_ 3*T*_h_ 1*T*_h_ − * − * − *
Sensor node	3*T*_h_ 2*T*_h_ 2*T*_h_ 4*T*_h_ 3*T*_h_ 1*T*_h_ − * − * − *
Total	24*T*_h_ 34*T*_h_ 35*T*_h_ 19*T*_h_ 28*T*_h_ 8*T*_ecc_+9*T*_h_
【*Communication cost*】	
Transmitted message	4 6 6 4 4 3 4 4 3

* Khan et al. scheme, Chen et al. scheme and Das scheme do not provide the key agreement phase for session key agreement.

Our proposed scheme uses only the hash function and XOR operations to design a simple authentication and key agreement scheme. However, the Yeh et al. scheme [8] provides a authentication and key agreement scheme which is established by an asymmetric encryption algorithm (specifically, an ECC). According to an experimental finding obtained in a related study, the one-way hash function is computationally efficient. The time complexity of the hash function is less than that of an asymmetric ECC encryption operation [2, 3, 7, 34, 35]. The following is a practical example for the computational costs: In an environment with a CPU of 3.2 GHz and with 3.0 GB of RAM, completing a one-way hash operation requires 0.02 ms on average when using SHA-1, and completing an asymmetric ECC encryption operation requires 0.45 ms on average when using ECC-160 [7].

For the user in each scheme run, the Yeh et al. scheme requires 0.94 ms for 2*T*_ecc_ + 2*T*_h_. The Amin et al. scheme requires 0.28 ms for 14*T*_h_. The Ostad-Sharif et al. scheme requires 0.24 ms for 12*T*_h_. By contrast, our scheme can perform the run in only 0.14 ms for 7*T*_h_. Therefore, the computational load of the user in the proposed scheme is reduced to 14.89% compared with the Yeh et al. scheme and to 58.33% compared with the Ostad-Sharif et al. scheme.

For the GWN in each scheme run, the Yeh et al. scheme requires 1.88 ms for 4*T*_ecc_ + 4*T*_h_. The Amin et al. scheme requires 0.34 ms for 17T_h_. The Ostad-Sharif et al. scheme requires 0.34 ms for 17T_h_. By contrast, our scheme can perform the run in only 0.22 ms for 11*T*_h_. Therefore, the computational load of the GWN in the proposed scheme is reduced to 11.7% compared with the Yeh et al. scheme and to 64.7% compared with the Ostad-Sharif et al. scheme.

For the sensor node in each scheme run, the Yeh et al. scheme requires 0.96 ms for 2*T*_ecc_ + 3*T*_h_. The Amin et al. scheme requires 0.08 ms for 4T_h_. The Ostad-Sharif et al. scheme requires 0.1 ms for 5T_h_. By contrast, our scheme can perform the run in 0.12 ms for 6*T*_h_. Therefore, the computational load of the sensor node in the proposed scheme is reduced to 12.5% compared with the Yeh et al. scheme.

**Fig 6:**
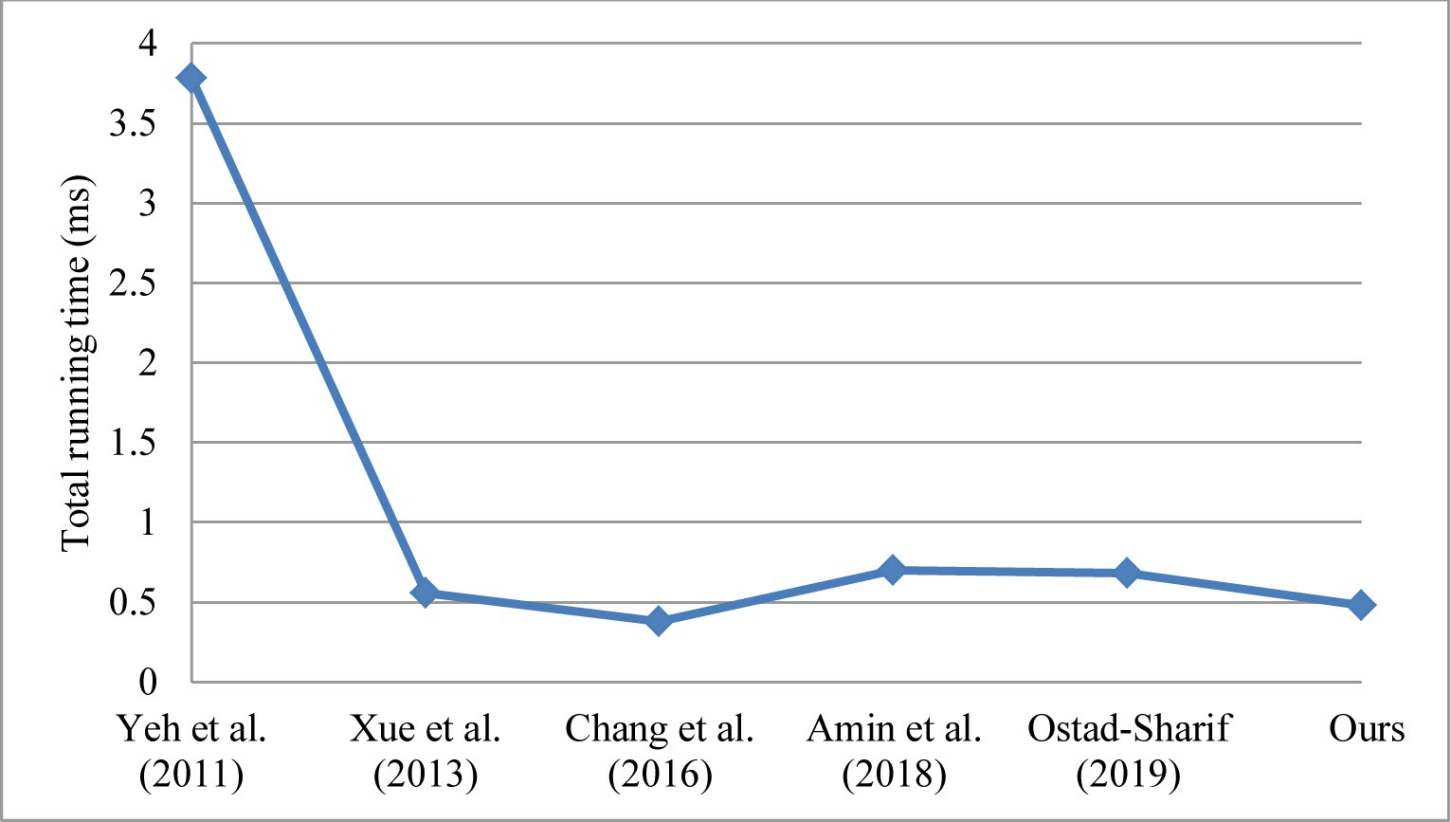
Comparison of running time.

In Table 4, the total computational costs of the schemes of Yeh et al., Xue et al., Chang et al., Amin et al., Ostad-Sharif et al., and ours are 8*T*_ecc_+9*T*_h_, 28*T*_h_, 19*T*h, 35*T*h, 34*T*h, and 24*T*h, respectively. Therefore, the total running time of the schemes of Yeh et al., Xue et al., Chang et al., Amin et al., Ostad-Sharif et al., and ours are 3.78, 0.56, 0.38, 0.7, 0.68, and 0.48 ms, respectively (Fig 6). Therefore, the total running time of our scheme is 12.7%, 85.7%, 68.6%, and 70.6% of that of the schemes of Yeh et al., Xue et al., Amin et al., and Ostad-Sharif et al., respectively. Although the total running time of our scheme (0.48 ms) is slightly greater than that of the Chang et al. scheme (0.38 ms), our scheme can overcome the security weaknesses of previous related schemes and provide greater security functionality (Table 3).

**Fig 7:**
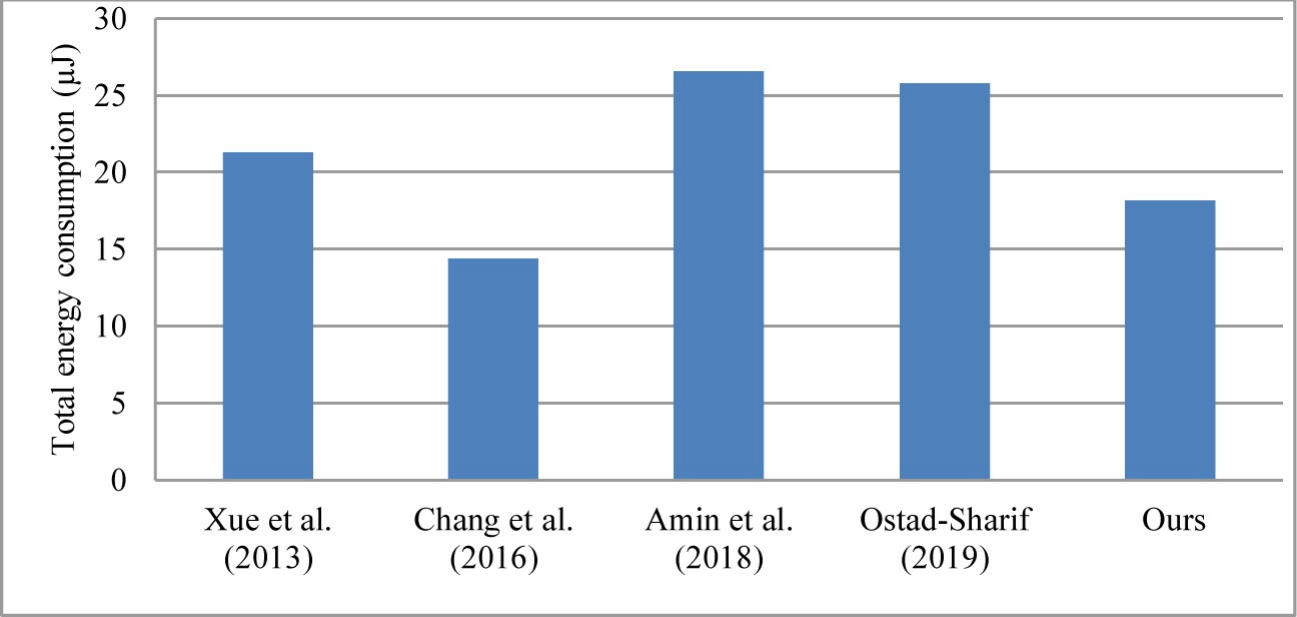
Comparison of energy consumption.

The energy consumption of the Yeh et al. scheme [8] is ascribed chiefly to the asymmetric ECC cryptosystem and hash functions. By contrast, the energy consumption of our scheme is principally attributed to the hash functions. As mentioned, the energy consumption for executing the hash function is much lower than that for executing an asymmetric ECC cryptosystem [38, 39]. A practical example follows: While using SHA-1 to compute the hash value, a 1-byte data packet requires 0.76 μJ of energy [43, 38, 39]. Nevertheless, a 163-bit ECC asymmetric cryptosystem requires 134.2 mJ of energy [38, 39]. As previously discussed, the total computational costs of the schemes of Yeh et al., Xue et al., Chang et al., Amin et al., Ostad-Sharif et al., and ours are 8*T*_ecc_+9*T*_h_, 28*T*_h_, 19*T*_h_, 35*T*_h_, 34*T*_h_, and 24*T*_h_, respectively (Table 4). Consequently, the total energy consumption levels of the schemes of Yeh et al., Xue et al., Chang et al., Amin et al., Ostad-Sharif et al., and ours are 1073606.8, 21.3, 14.4, 26.6, 25.8, and 18.2 μJ, respectively. Consequently, in each scheme run, the total energy consumed by our scheme is 0.0017%, 85.4%, 68.4%, and 70.5% of that consumed by the schemes of Yeh et al., Xue et al., Amin et al., and Ostad-Sharif et al., respectively (Fig 7). Because the total energy consumption of the Yeh et al. scheme is excessive relative to other schemes, it cannot be shown in Fig 7. Although the total energy consumption of our scheme (18.2 μJ) is slightly greater than that of the Chang et al. scheme (14.4 μJ), our scheme provides superior security functionality to overcome the weaknesses of previous schemes (Table 3).

As mentioned, the communication cost accounts for the number of messages transmitted. A low number of transmitted messages results in less consumption for the message overhead [9, 42]. In completing the authentication and key agreement phase, the total numbers of transmitted messages of the schemes of Ostad-Sharif et al., Amin et al., Chang et al., Xue et al., Yeh et al., and ours are 6, 6, 4, 4, 3, and 4, respectively (Table 4). Although the communication costs of the proposed scheme (4 transmitted messages) is slightly greater than the Yeh et al. scheme (3 transmitted messages), the Yeh et al. scheme is subject to high computational costs (3.78 ms, Fig 6) and large energy consumption (1073606.8 μJ) due to its use of ECC.

In this subsection, we demonstrate that our scheme is highly efficient because of the superior performance: low computational cost (0.14 ms for the user, 0.12 ms for the sensor node, and 0.22 ms for the GWN), low energy consumption (18.2 μJ for the authentication and key agreement phase), and low communication cost (4 transmitted messages for the authentication and key agreement phase, 0 transmitted messages for the password change phase).

## 6. Conclusions

This paper analyzes the security weaknesses of related authentication schemes and proposes a more efficient and secure authentication scheme for WSNs in IoT environments. The BAN logic method is used to prove our scheme. Finally, we compare the functional effectiveness and performance efficiency of our scheme with those of previously published schemes. Cryptanalysis revealed that our scheme overcomes the security weaknesses of the previously published schemes. Our scheme satisfies the requirement of basic design criteria for the authentication scheme as well. Consequently, our scheme can enhance security effectiveness in real-world IoT environments and provide additional security functionalities compared with the other discussed schemes. Moreover, performance analysis revealed that our scheme demonstrates high efficiency and superior performance.

Our future work and challenges include attempting to find security risks in heterogeneous IoT environments. Various heterogeneous IoT applications can cause serious challenges in securing networks. Future studies will further evaluate the reliability and scalability of the proposed scheme in heterogeneous IoT environments. Moreover, we also study highly secure machine learning-based authentication schemes for WSNs in intelligent IoT environments. The integration of Big Data with intelligent IoT networks will be challenging due to the limited resources of WSNs.
