# Correction: Efficient and secure three-party mutual authentication key agreement protocol for WSNs in IoT environments

**DOI:** 10.1371/journal.pone.0234631

**Published:** 2020-06-12

**Authors:** Chi-Tung Chen, Cheng-Chi Lee, Iuon-Chang Lin

There are errors in the author affiliations. The correct affiliations are as follows:

Chi-Tung Chen^1^, Cheng-Chi Lee^3,4^, Iuon-Chang Lin^2,4^

**1** Department of Distribution Management (Information Management), National Chin-Yi University of Technology, Taichung, Taiwan, R.O.C. **2** Department of Library and Information Science, Research and Development Center for Physical Education, Health, and Information Technology, Fu Jen Catholic University, New Taipei City, Taiwan, R.O.C. **3** Department of Photonics and Communication Engineering, Asia University, Taichung, Taiwan, R.O.C. **4** Department of Management Information Systems, National Chung Hsing University, Taichung, Taiwan, R.O.C.

There is an error in Fig 4. Part of the figure is missing. Please see the complete, correct Fig 4 here.

**Fig 4:**
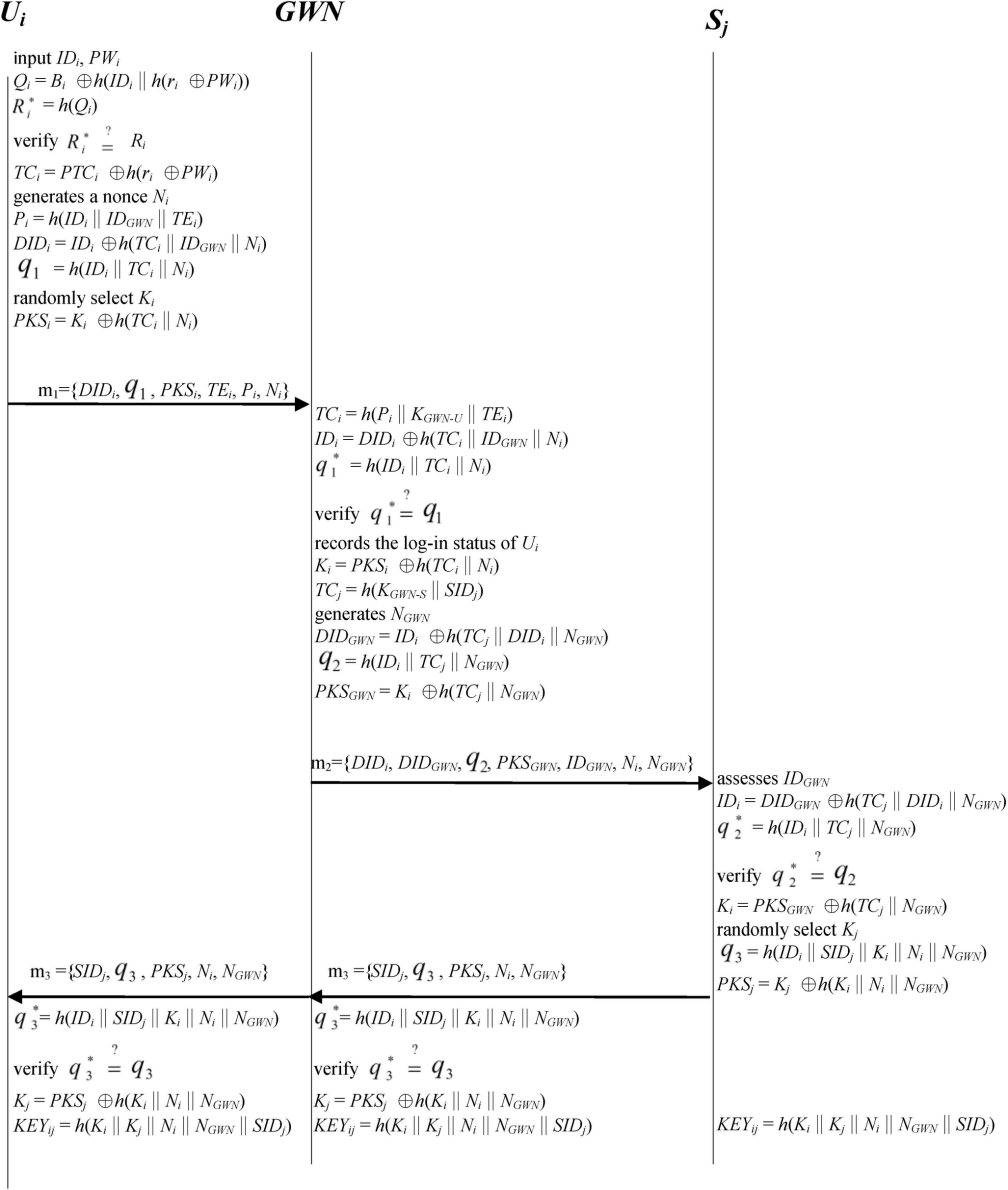
Login phase; authentication and key agreement phase.

There are errors in the typesetting of the columns in Tables 3 and 4. Please see the correct Tables 3 and 4 here.

**Table 3:** Functionality comparison of our scheme with other related schemes.

	Ours	Ostad-Sharif (2019)[2]	Amin et al. (2018)[26]	Chang et al. (20160[27]	Xue et al. (2103)[7]	Yeh et al. (2011)[8]	Khan et al. (2010)[24]	Chen et al. (2010)[25]	Das (2009)[5]
Password protection	Yes	Yes	Yes	No	No	Yes	Yes	No	No
Stolen smart card attack resistance	Yes	Yes	Yes	No	No	No	No	No	No
Masquerade attack resistance	Yes	Yes	Yes	No	Yes	Yes	Yes	No	No
Replay attacks resistance	Yes	Yes	Yes	No	Yes	No	Yes	Yes	Yes
Insider attack resistance	Yes	Yes	Yes	Yes	No	Yes	Yes	No	No
Password updating/changing	Yes	No	Yes	Yes	No	No	Yes	No	No
Time synchronization avoidance	Yes	No	No	No	No	Yes	No	No	No
Mutual authentication	Yes	No	Yes	Yes	Yes	Yes	Yes	Yes	No
Session key agreement	Yes	Yes	Yes	Yes	Yes	Yes	No	No	No
User anonymity	Yes	Yes	Yes	No	Yes	No	Yes	Yes	Yes
GWN bypassing attack resistance	Yes	Yes	Yes	Yes	Yes	Yes	No	No	No

**Table 4:** Performance comparison of our scheme with other related schemes.

	Ours	Ostad-Sharif (2019)[2]	Amin et al. (2018)[26]	Chang et al. (20160[27]	Xue et al. (2103)[7]	Yeh et al. (2011)[8]	Khan et al. (2010)[24]	Chen et al. (2010)[25]	Das (2009)[5]
【*Computational cost*】									
*Authentication phase*									
User	4*T*_h_	10*T*_h_	13*T*_h_	3*T*_h_	5*T*_h_	2*T*_ecc_*+*1*T*_h_	3*T*_h_	4*T*_h_	3*T*_h_
GWN	8*T*_h_	14*T*_h_	14*T*_h_	5*T*_h_	11*T*_h_	4*T*_ecc_*+*3*T*_h_	5*T*_h_	5*T*_h_	4*T*_h_
Sensor Node	3*T*_h_	3*T*_h_	2*T*_h_	1*T*_h_	3*T*_h_	2*T*_ecc_*+*2*T*_h_	2*T*_h_	2*T*_h_	1*T*_h_
*key agreement phase*									
User	3*T*_h_	2*T*_h_	1*T*_h_	3*T*_h_	3*T*_h_	1*T*_h_	- ^*^	- ^*^	- ^*^
GWN	3*T*_h_	3*T*_h_	3*T*_h_	3*T*_h_	3*T*_h_	1*T*_h_	- ^*^	- ^*^	- ^*^
Sensor Node	3*T*_h_	2*T*_h_	2*T*_h_	4*T*_h_	3*T*_h_	1*T*_h_	- ^*^	- ^*^	- ^*^
Total	24*T*_h_	34*T*_h_	35*T*_h_	19*T*_h_	28*T*_h_	8*T*_ecc_+9*T*_h_			
【*Communication cost*】									
Transmitted message	4	6	6	4	4	3	4	4	3

^*^ Khan et al. scheme, Chen et al. scheme and Das scheme do not provide the key agreement phase for session key agreement.
